# Integrated control of cancer stemness by σ_1_ receptor in advanced prostate cancer

**DOI:** 10.1038/s41388-025-03541-7

**Published:** 2025-09-02

**Authors:** Gianluca Civenni, Giada Sandrini, Jessica Merulla, Carola Musumeci, Elisa Federici, Arianna Vallegra, Aleksandra Kokanovic, Simone Mosole, Dheeraj Shinde, Elisa Sorrenti, Alyssa J. J. Paganoni, Martina Marchetti, Riccardo Valzelli, Domenico Albino, Matteo Pecoraro, Andrea Rinaldi, Marco Bolis, Roger Geiger, Tobias Winge, Catharina Holtschulte, Erik Laurini, Sabrina Pricl, Giuseppina M. Carbone, Bernhard Wünsch, Carlo V. Catapano

**Affiliations:** 1 Institute of Oncology Research (IOR), Università della Svizzera italiana (USI), Bellinzona, Switzerland; 2 Bioinformatics Core Unit, Institute of Oncology Research (IOR) and Swiss Institute of Bioinformatics, Bellinzona, Switzerland; 3 Mario Negri Institute for Pharmacological Research, Milano, Italy; 4 Institute for Research in Biomedicine (IRB), Università della Svizzera italiana (USI), Bellinzona, Switzerland; 5 Institut für Pharmazeutische und Medizinische Chemie, Westfälische Wilhelms-Universität Münster, Münster, Germany; 6 Molecular Biology and Nanotechnology Laboratory (MolBNL@UniTS), DEA, University of Trieste, Trieste, Italy

**Keywords:** Cancer stem cells, Targeted therapies

## Abstract

Cancer stem cells (CSCs) are pervasively present in human cancers and have a fundamental role in treatment failure and disease recurrence. Identifying critical elements that sustain the CSC phenotype may lead to novel strategies for cancer treatment. Here, we provide evidence of an essential link between the σ_1_ receptor (σ_1_R), a ligand-regulated chaperone protein residing preferentially at the endoplasmic reticulum-mitochondria contact sites, and CSCs in castration-resistant prostate cancers (CRPCs). Integrating functional assays in multiple preclinical models with transcriptomic and proteomic data, we found that σ_1_R controls CSC self-renewal capacity and tumorigenic proficiency by coordinating mitochondrial dynamics and mitochondrial-nuclear signaling. Inhibiting σ_1_R with synthetic antagonists and RNA interference led to the progressive exhaustion and loss of tumorigenicity of the CSC progeny. Mechanistically, interfering with σ_1_R function disrupted mitochondria homeostasis and triggered β-catenin degradation. Examining clinical CRPC samples, we found a tight correlation between σ_1_R and mitochondrial gene expression. Furthermore, σ_1_R and β-catenin protein levels were highly correlated in prostate tumors with significant upregulation in metastatic CRPCs, sustaining a role of the σ_1_R-mitochondria-β-catenin axis in disease progression. This σ_1_R-centered axis is essential for preserving the self-renewal and tumorigenic capability of CSCs and represents a critical vulnerability exploitable for discovering novel CSC-directed therapies.

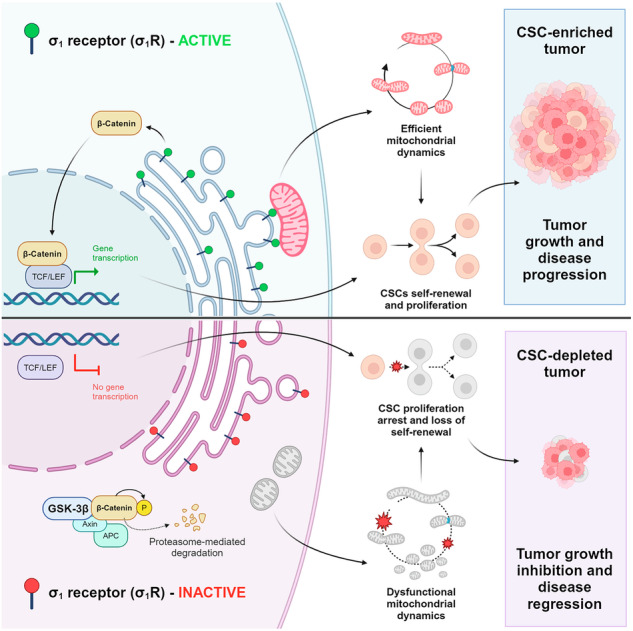

## Introduction

Cancer stem cells (CSCs) endowed with cardinal stem cell properties, like self-renewal and unlimited proliferative potential, contribute substantially to intra-tumor heterogeneity and play a fundamental role in tumor progression, treatment failure, and disease recurrence therapeutics [1–4]. CSCs are intrinsically resistant to most anticancer therapies, including cytotoxic drugs, molecular-targeted therapeutics, and immune therapies [1–5]. Identifying essential factors and critical signaling pathways that sustain the stem cell-like phenotype may lead to new approaches to eradicate CSCs and novel strategies for cancer treatment [6]. Canonical developmental and stem cell signaling pathways contribute to maintaining the proliferative potential of the CSC population and have provided actionable targets for restraining CSC expansion [6, 7]. At their core, CSCs exhibit an imbalance between differentiation and self-renewal, which favors the maintenance and continuous proliferation of the CSC population [8]. At the cellular level, CSCs undergo symmetric and asymmetric cell divisions to preserve and propagate indefinitely the progeny of daughter cells with intact stem cell properties [1, 2]. Recent studies converge on the notion that mitochondria play a critical role in pacing symmetric and asymmetric stem cell division and controlling the balance between stem cell differentiation and self-renewal [9–12]. Reciprocal mitochondria-nuclear communications may further coordinate cellular and mitochondrial divisions to ensure the correct mitochondria partitioning between daughter cells and unlimited propagation of the CSC progeny [9, 10]. Disruption of these signaling axes could alter the fine-tuned equilibrium sustaining self-renewal and proliferation of CSCs. Consequently, the factors involved in these processes might be relevant targets for promoting the selective elimination of CSCs.

The σ_1_R receptor (σ_1_R) is a unique 25-KDa integral transmembrane protein localized primarily at the endoplasmic reticulum (ER) [13]. σ_1_R has no sequence homology to any other human protein [14]. It comprises a short transmembrane domain and a large cytoplasmic domain containing the ligand binding pocket to accommodate small-molecule ligands [14–17]. σ_1_R ligands act as agonists or antagonists depending on their binding mode and ability to induce changes in the receptor conformation and oligomerization state [14–17]. σ_1_R functions as a ligand-operated chaperone, interacting with multiple proteins and controlling protein folding and stability in a cell-type and context-dependent manner [14, 16]. It resides preferentially at the ER-mitochondria contact points or mitochondria-associated ER membranes (MAMs), where it contributes to the MAM organization and regulation of ER-mitochondria signaling [14, 16]. Moreover, σ_1_R may act as an inter-organelle signaling modulator, integrating signals between the ER, mitochondria, and nucleus and taking part in numerous physiological and pathological processes [18, 19]. σ_1_R has been studied extensively in the context of neurodegenerative disorders, and synthetic σ_1_R ligands have been tested primarily in these disease models [20, 21]. σ_1_R is upregulated in human cancers and has been implicated in tumor development and therapy [22–25]. However, the impact of σ_1_R and the effects of σ_1_R modulators in cancer are still poorly defined [22].

Here, we provide evidence of a selective link between σ_1_R function and the ability of CSCs to self-renew, expand, and generate tumors. Moreover, we found that inhibiting σ_1_R with synthetic antagonists and genetic knockdown led to the progressive exhaustion and loss of self-renewal and tumorigenic potential of the CSC progeny in multiple preclinical models of prostate cancer. This study, hence, defines a σ_1_R-centered axis involving mitochondrial homeostasis and mitochondrial-nuclear signaling, which is essential for preserving the self-renewal capability and continuous expansion of tumor-initiating stem-like cells and represents a critical vulnerability exploitable for therapeutic targeting of CSCs in human cancers.

## Materials and methods

### Cell lines, key reagents, preclinical models, and clinical samples

Cell lines were obtained from the American Type Culture Collection (ATCC), authenticated by STR profiling, and maintained according to the provider’s indications. Cells were checked regularly for *Mycoplasma* contamination (MycoAlert Mycoplasma detection kit, Lonza). The synthesis of WMS 26-09, WMS 26-10, and WMS 26-02 were described previously [26, 27]. The compounds were dissolved in DMSO (10 mM) and stored at −20 °C. All studies involving mice were received ethical approval from the Swiss Veterinary Authority (license 32174; TI 04/2020). Male 4-week-old athymic nude mice purchased from Invigo were used to establish subcutaneous xenograft models with the indicated cell lines. NOD.Cg-*Prkdc*^*scid*^ *Il2rg*^*tm1WjI*^/SzJ (NSG) male mice were purchased from Charles River Laboratories and used to maintain patient-derived xenografts (PDXs) by serial passaging. Transgenic/knockout *Pb-Cre4;Pten*^*flox/flox*^*;R26*^*ERG*^ (ERG^+^/PTEN^-^) mice with combined prostate-specific deletion of PTEN and overexpression of ERG [28] were maintained in-house and used to generate ex vivo tumor spheres and organoids and in vivo experiments. Murine EPG2 prostate cancer cells were derived from ERG^+^/PTEN^-^ mouse prostate tumors to perform in vitro studies. Archival formalin-fixed paraffin-embedded tissue samples from patients with primary prostate cancer and hormone-refractory metastatic tumors were obtained upon informed consent and ethical approval by the Multimedica Hospital Institutional Ethical Committee (Milan, Italy). A published collection of integrated RNA-seq data from multiple studies was used to investigate σ_1_R levels and global gene expression profiles in clinical samples [29]. All experiments and methods were performed in accordance with the relevant guidelines and regulations. For additional information on the study methods and materials, see the detailed description and the Reagents and Resources Table provided in the Supplemental Information file available online.

## Results

### σ_1_R promotes self-renewal and tumor-initiating capability of prostate cancer stem cells

**Fig. 1:**
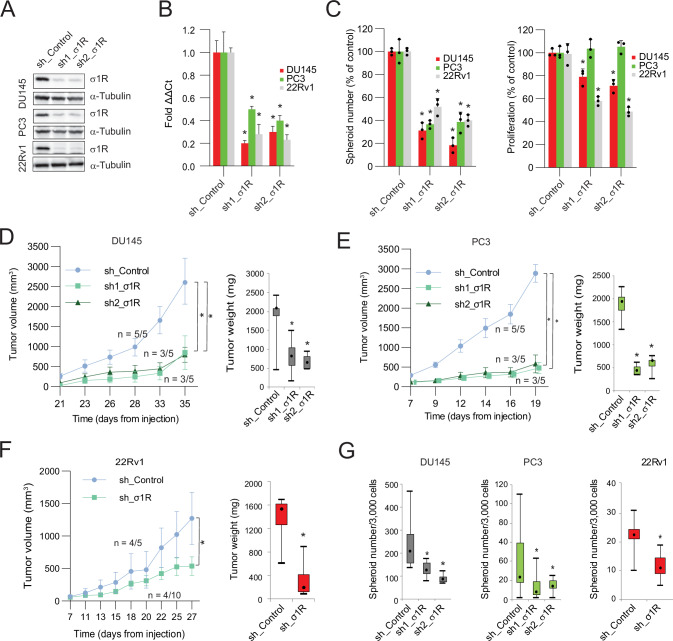
σ_1_R controls stemness and tumorigenicity of prostate cancer cells. σ_1_R knockdown by shRNAs in DU145, PC3 and 22Rv1 cells by immunoblotting (**A**) and qRT-PCR (**B**). **C** Tumor-sphere formation and proliferation of σ_1_R-depleted (sh1_σ1R, sh2_σ1R) and control (sh_Control) cells. **D–F** Growth and weight of xenografts of σ_1_R-depleted and control DU145, PC3 and 22Rv1 cells. Data are mean ± SEM. Number of mice developing tumors and total number of mice injected per group are indicated. **G** Ex vivo tumor-sphere growth from control and σ_1_R-depleted DU145, PC3 and 22Rv1 xenografts. For 22Rv1 xenografts data with sh1 and sh2_σ1R shRNA are combined. Boxplots represent median, interquartile range, maximum and minimum. Data are mean ± SD; *P < 0.01 by *t-*test and ANOVA.

To investigate the impact of σ_1_R in prostate cancer, we generated human prostate cancer cell lines with stable knockdown of σ_1_R by transducing lentiviral short hairpin RNA (shRNA) constructs. These cell lines represent castration-resistant models that are androgen receptor (AR) negative (DU145, PC3) or express an AR variant (22Rv1). We verified the efficiency of σ_1_R silencing by immunoblotting and qRT-PCR (Fig. 1A, B). Using these control and σ_1_R-silenced cellular models, we set out to compare the consequences of σ_1_R depletion on the bulk heterogeneous population of cancer cells and the cancer stem-like cell population with tumor-initiating and self-renewal properties. In these studies, we relied on the unique ability of cancer stem-like cells to grow as 3D tumor spheres in stringent, stem-selective culture conditions (i.e., non-adherence, serum-free medium, low cell density) to isolate CSCs from bulk tumor cell cultures [8]. Tumor sphere-forming ability is a common feature shared by cancer stem-like cells of different origins [8]. Instead, tumor cells in the bulk population devoid of stem-like properties do not proliferate and die in non-adherent growth cultures. Importantly, this approach is unbiased and does not rely on any prior knowledge of specific stem cell surface markers, which can vary widely in samples from different sources [30]. We have shown previously that tumor sphere-forming cells from various sources retain stemness properties and propagate for multiple generations (self-renewal capability) when maintained in stem cell-selective culture conditions [31–34]. They consistently express typical stem cell genes and have greatly enhanced in vivo tumor-initiating ability. Thus, the tumor-sphere assay probes the ability of cancer stem-like cells present within the bulk tumor cell population to expand and form single stem cell-derived 3D colonies in non-adherent conditions and, hence, reflects their self-renewal and proliferative capability. We found that the knockdown of σ_1_R substantially reduced tumor-sphere growth and had a more modest effect on the proliferation of bulk tumor cells in 2D cultures (Fig. 1C), indicating a predominant impact of σ_1_R depletion on the tumor-initiating stem-like cell population.

Next, we tested whether σ_1_R depletion affected the tumor-initiating capability of stem-like cancer cells in vivo by implanting control and σ_1_R-depleted cancer cells in immunodeficient mice. Impaired function and reduced numbers of stem-like tumor cells within the bulk population of tumor cells would substantially impact tumor number and growth in vivo [31–34]. We found that σ_1_R depletion impaired the tumor-initiating ability (i.e., fewer tumors per mouse) and expanding capability (i.e., delayed tumor growth) of stem-like cancer cells, consistent with a reduction of proficient CSCs (Fig. 1D–F). We also quantified in ex vivo tumor sphere-forming assays the pool of stem-like cancer cells in the control and σ_1_R-depleted tumor xenografts. Tumors of σ_1_R-depleted cells had a drastically reduced content of stem-like cancer cells, confirming the persistent impairment of CSC proliferation and expansion imposed by σ_1_R inactivation (Fig. 1G). Thus, σ_1_R was required to preserve the pool of tumor-initiating stem-like cells both in vitro and in vivo. These results provided a new paradigm for σ_1_R function in prostate cancer and suggested the use of σ_1_R antagonists for targeting stem-like cancer cells. Blocking σ_1_R would push CSCs into a state of reduced self-renewal, proliferative, and tumor-initiating capability.

### σ_1_R antagonists block cancer stem cell expansion

**Fig. 2:**
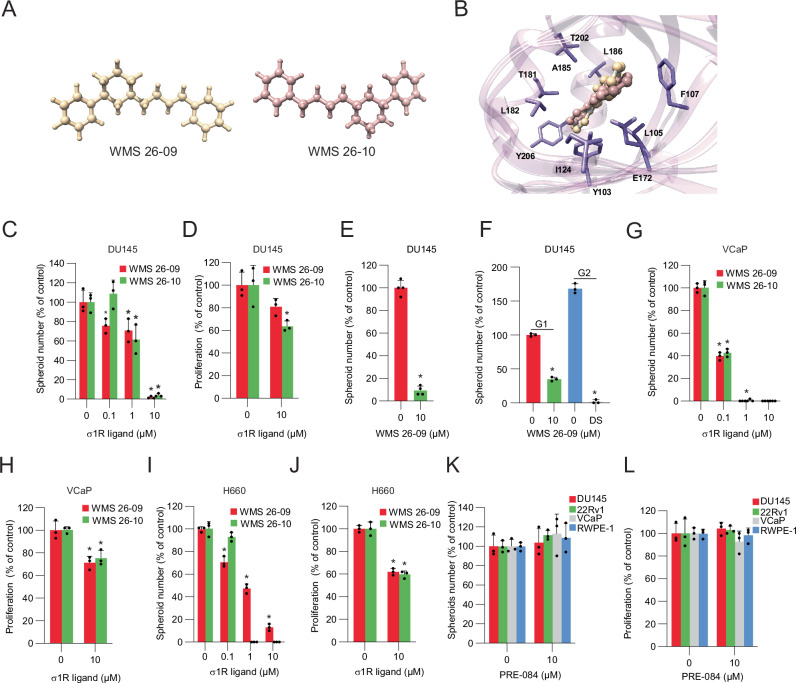
σ_1_R antagonists impair cancer stem cell expansion in vitro. **A** Chemical structures of WMS 26-09 and WMS 26-10. Compounds are shown as colored sticks-and-ball structures. **B** Overlay of WMS 26-09 (tan) against WMS 26-10 (rosy brown) binding to σ_1_R. The structure of σ1R was obtained from the RCSB Protein Data Bank (PDB ID 5HK1). Estimated free energy of binding (ΔG_bind_) for WMS 26-09 and WMS 26-10 by the Molecular Mechanics/Poisson Boltzmann Surface Area (MM/PBSA) approach were −11.22 kcal/mol and −10.76 kcal/mol, respectively. Tumor-sphere (**C**) and proliferation (**D**) of DU145 cells incubated with σ_1_R antagonists WMS 26-09 and WMS 26-10. **E** Tumor-sphere formation of pre-selected DU145 stem-like cells treated with WMS 26-09. **F** Tumor-sphere formation by DU145 cells treated with WMS 26-09 (10 µM) during the first generation (G1) and after replating for the second generation (G2) in drug-free medium. **G**, **H** Tumor-sphere formation (**G**) and proliferation (**H**) of VCaP cells treated with σ_1_R antagonists. **I**, **J** Tumor-sphere formation (**I**) and proliferation (**J**) of H660 cells treated with σ_1_R antagonists. **K**, **L** Tumor-sphere and prostate-sphere formation (**K**) and proliferation (**L**) of DU145, 22Rv1, VCaP, and RWPE-1 exposed to PRE-084 (10 µM). Data are mean ± SD; *P < 0.01 by *t-*test and ANOVA.

Synthetic ligands modulate the σ_1_R function and affect downstream pathways and biological processes in a cell context-dependent manner [14, 16]. We selected two recently described σ_1_R antagonists, WMS 26-09 with the (*1R,3S*)-configuration and its enantiomer WMS 26-10 with the (*1S,3R*)-configuration (Fig. 2A). Both compounds, along with their racemic mixture (WMS 26-02), had remarkably high affinity for σ_1_R with K_i_ of about 1 nM in biochemical assays [26]. In silico molecular dynamics simulation using the crystal structure of human σ_1_R [15] revealed that the two compounds make similar interactions and multiple favorable contacts with residues in the ligand-binding pocket, affording the compounds high binding affinity (Fig. 2B). When tested in cellular assays, the σ_1_R antagonists significantly inhibited the growth of DU145 tumor spheres (Fig. 2C), reflecting the consequences of σ_1_R genetic silencing. Still, they had a limited effect on the proliferation of bulk tumor cells (Fig. 2D). Importantly, we saw similar effects in experiments in which we pre-selected stem-like cancer cells and then incubated them in the presence or absence of σ_1_R ligands (Fig. 2E). The results showed significantly reduced growth of pre-selected CSCs, similar to non-selected CSCs from bulk cell cultures, thus confirming that they were the critical cell population targeted by σ_1_R inhibition. To test further the impact on CSC self-renewal, we recovered tumor spheres after exposure to the vehicle or a σ_1_R antagonist at the first generation (G1). Then, we plated equal numbers of viable stem-like cells in the drug-free medium to determine the number of tumor spheres at the second generation (G2). The vehicle-treated cells reformed tumor spheres at G2 with similar or even higher efficiency, as expected (Fig. 2F). Conversely, the stem-like cancer cells exposed to the σ_1_R ligand at G1 formed a drastically reduced number of tumor spheres at G2, indicative of a substantial and persistent decline in their self-renewal and ability to propagate in 3D stem cell-selective conditions. We observed similar results in PC3 and 22Rv1 cells. In both cell lines, σ_1_R antagonists reduced the growth of the tumor spheres but were substantially less effective on bulk tumor cell proliferation (Supplementary Fig. S1A, B). Interestingly, antagonists of the σ_1_R receptor reduced both the number and the size of tumor spheres, indicating an effect on the ability of CSCs to self-renew, expand, and sustain the continuous propagation of the CSC population (Supplementary Fig. S1C–E).

To verify the preferential vulnerability of CSCs in other models, we tested the σ_1_R antagonists in additional prostate cancer cell lines expressing σ_1_R (Supplementary Fig. S1F). VCaP and NCI-H660 cells are CRPC models having AR amplification and AR silencing, respectively. These cells have the TMPRSS2:ERG gene fusion, the most common genetic rearrangement found in human prostate cancers [35]. σ_1_R antagonists very effectively suppressed tumor sphere growth with significant inhibition at ≤1 µM (Fig. 2G–J). Again, the impact on the proliferation of tumor cells in 2D cultures was significantly less, even at 10 µM of ligand concentration. Interestingly, the σ_1_R agonist PRE-084 did not affect the cell proliferation, tumor spheres, or prostate sphere formation in cancer cell lines and normal prostate epithelial RWPE-1 cells (Fig. 2K, L). Antagonists, as well as genetic σ_1_R knockdown, also had a modest impact on proliferation and prostate sphere formation by RWPE-1 cells (Supplementary Fig. S1G–J). Thus, σ_1_R inhibition had a potent impact preferentially on the growth of tumor spheres, indicating a critical requirement for σ_1_R in the CSC subpopulation in multiple human prostate cancer cell lines.

### σ_1_R antagonists arrest the growth of patient- and mouse-derived tumor spheres and organoids

**Fig. 3:**
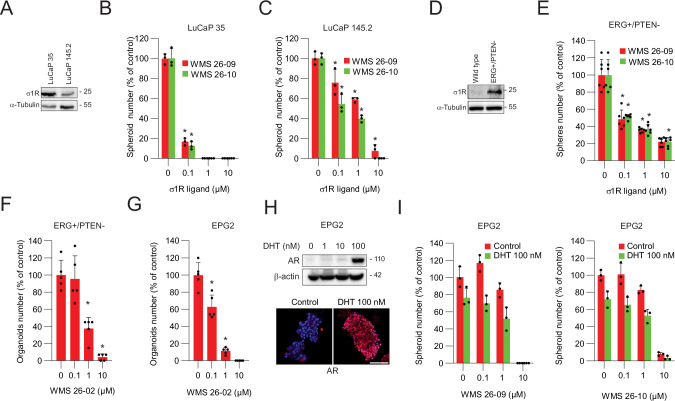
Impact of σ_1_R inhibition on tumor spheres and organoids from patient- and transgenic mouse-derived models. **A** σ_1_R expression in LuCaP 35 and LuCaP 145.2 patient-derived xenografts. **B**, **C** Tumor-sphere formation of LuCaP 35 and LuCaP 145.2 derived tumor cells treated with σ_1_R antagonists. **D** σ_1_R expression in prostatic tissue from wild type and ERG^+^/PTEN^-^ transgenic mice. **E** Growth of ERG^+^/PTEN^-^ derived tumor-spheres treated with σ_1_R antagonists. Growth of tumor organoids derived ERG^+^/PTEN^−^ tumors (**F**) and EPG2 cells (**G**) treated with WMS 26-02. **H** Androgen receptor (AR) expression in 2D cultures (top) and 3D tumor sphere cultures (bottom) of EPG2 cells exposed to DHT. **I** Tumor-sphere formation of EPG2 cells incubated with σ_1_R antagonists with or without DHT. Data are mean ± SD; *P < 0.01 by *t-*test and ANOVA.

To investigate further the effects of σ_1_R antagonists, we generated ex vivo tumor spheres from two patient-derived xenografts (PDXs), LuCaP 35 and LuCaP 145.2, which both expressed σ_1_R (Fig. 3A). LuCaP 35 is a TMPRSS2:ERG fusion-positive and AR-positive PDX model, whereas LuCaP 145.2 is AR-negative and has a neuroendocrine phenotype [36]. Tumor sphere-forming assays allowed the functional isolation of cancer stem-like cells with typical stemness properties and high tumor-initiating capability from PDX models [37]. Consistent with the stem-like phenotype, tumor sphere-forming cells from LuCaP 35 and LuCaP 145.2 injected in NSG mice generated tumors with much higher efficiency than bulk tumor cells (10^4^ vs. 10^6^ tumor cells/mouse), demonstrating their high in vivo tumorigenicity (Supplementary Fig. S2). WMS 26-09 and WMS 26-10 inhibited the growth of tumor spheres of both PDX models very effectively (Fig. 3B, C). We used a similar approach to isolate cancer stem-like cells from prostate adenocarcinoma derived from the *Pb-Cre4;Pten*^*flox/flox*^*;R26*^*ERG*^ (ERG^+^/PTEN^-^) mice and examine the effects of σ_1_R antagonists. The ERG^+^/PTEN^-^ mice combine the prostate-specific knock-in of the TMPRSS2:ERG gene fusion and the deletion of the tumor suppressor PTEN and develop highly invasive prostate adenocarcinoma [28, 38], in which σ_1_R is overexpressed (Fig. 3D). ERG^+^/PTEN^-^-derived prostate tumor cells plated ex vivo in non-adherent and stem cell-selective conditions form tumor spheres enriched in cancer stem-like cells that retain stemness and tumor-initiating properties and rapidly form tumors in mice [39]. Notably, σ_1_R antagonists strongly inhibited the growth of the ERG^+^/PTEN^-^-derived tumor spheres (Fig. 3E), indicating that murine prostate CSCs in this model were susceptible to σ_1_R inhibition.

We further tested the impact of σ_1_R antagonists in 3D tumor organoids generated from ERG^+^/PTEN^-^-derived tumor cells and a murine cell line, EPG2 cells, established from ERG^+^/PTEN^-^ prostate tumors. In the organoid assay, stem-like cancer cells embedded in a semi-solid extracellular matrix (Matrigel) expand, proliferate, and partially differentiate to regenerate the initial heterogeneous tumor cell population and reproduce morphologically and phenotypically the 3D structure of the original tumor [8]. Hence, the 3D organoid assays can reveal the effects on the stem-like cancer cells and their tumorigenic phenotype. Remarkably, the σ_1_R antagonist WMS 26-02 was very effective in blocking the growth of organoids formed by the primary ERG^+^/PTEN^-^ tumor cells and the EPG2 cell line (Fig. 3F, G). Interestingly, EPG2 cells retain a progenitor/stem-like phenotype and a high degree of phenotypic plasticity between a luminal and non-luminal state. Indeed, exposure to DHT can shift EPG2 cells from a predominant AR^negative^ to an AR^positive^ state (Fig. 3H). Taking advantage of this feature, we investigated whether AR signaling could contribute to the response of prostate cancer cells to σ_1_R antagonists. Treatment with σ_1_R antagonists inhibited tumor sphere formation (Fig. 3I). However, the addition of DHT (100 nM) did not modify the response of tumor sphere-forming cells to the σR antagonists, showing that the effects on cancer stem-like cells were independent of AR signaling. This finding is in line with the predominant AR^low/negative^ and androgen-independent phenotype of prostate CSCs in preclinical models.

### σ_1_R inhibition causes complex transcriptomic and proteomic changes

**Fig. 4:**
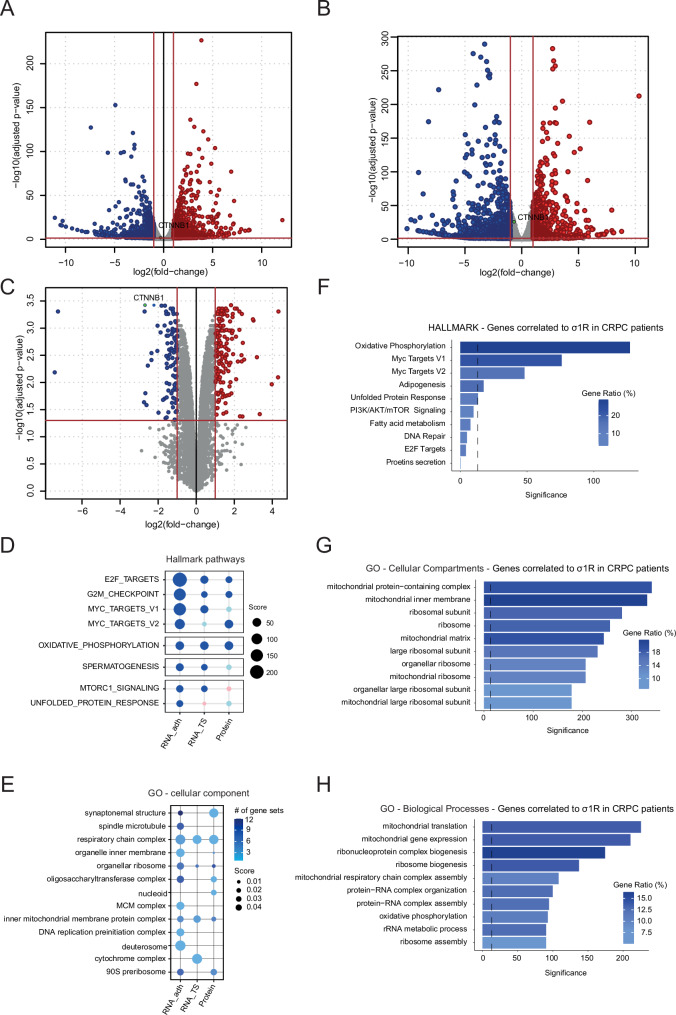
σ_1_R influences transcriptional regulation and mitochondrial function. Transcriptomic changes in DU145 bulk tumor cells (**A**) and tumor spheres (**B**) after σ_1_R knockdown. Thresholds for adjusted p-value and |log_2_(FC)| are 0.05 and 1, respectively. **C** Proteomic changes in σ_1_R-depleted DU145 cells. Down-regulated Hallmark pathways (**D**) and Cellular Components Ontology terms (**E**) in σ_1_R-depleted cells from transcriptomic (RNA_adh, RNA_TS) and proteomic (Prot_adh) analysis. **F** Barplot representing gene set enrichment analysis (GSEA) of Hallmark pathways based on pre-ranked Pearson’s correlation coefficients between global gene expression and σ_1_R expression levels. GSEA analyses on Gene Ontology terms (**G** Cellular components; **H** Biological processes) based on pre-ranked Pearson’s correlation coefficients between gene expression and σ_1_R expression levels. The colors represent the enrichment scores, while the length of the bars represents false discovery rates. The bold dotted intercept represents a logarithmically transformed FDR threshold of 0.05.

Our data uncovered a link between σ_1_R and CSC self-renewal in CRPC models. To get insights into the underlying mechanisms, we performed RNA-sequencing (RNA-seq) in 2D adherent cultures and 3D CSC-enriched tumor spheres of control and σ_1_R-depleted DU145 cells. Several genes were up and down-regulated (*p* ≤ 0.05, |log2FC|>1) after σ_1_R knockdown in bulk and tumor sphere-forming cells (Fig. 4A, B and Supplementary Dataset S1, S2). The proteomic analysis also showed relevant changes upon σ_1_R depletion (Fig. 4C and Supplementary Dataset S3). Strikingly, the two cell populations shared a substantial amount of differentially expressed transcriptional and proteomic features (Supplementary Fig. S3). To functionally assess the impact of the σ_1_R knockdown, we compiled a list of stem cell marker genes by assembling an adult stem cell (ASC) signature [40] and a broader set of stem cell markers studied in prostate cancer [41]. Using the RNA-seq data, we found significant downregulation of many stem cell-related genes both in bulk and tumor-sphere cells after σ_1_R knockdown (Supplementary Fig. S4), confirming a relevant impact of the stem cell-like phenotype. Functional annotation and enrichment analysis further revealed that the hallmark pathways predominantly downregulated concomitantly with σ_1_R knockdown were E2F and MYC targets, G2/M checkpoints, and oxidative phosphorylation in both transcriptomic and proteomic analyses, thus pointing to transcriptional regulation and mitochondrial function as the main processes affected (Fig. 4D). Among the GO-cellular components, we also observed a prevalence of mitochondrial elements (e.g., respiratory chain complex, inner mitochondrial membrane proteins) across the transcripts and proteins down in σ_1_R-depleted cells (Fig. 4E).

To evaluate the relevance of these findings beyond our preclinical model, we looked at genes highly correlated with σ_1_R in an extensive compendium of RNA-seq data from prostate cancer patients, including 664 primary tumors and 249 CRPCs [29]. We found several genes positively correlated with σ_1_R in the CRPC samples and, conversely, very few genes significantly correlated to σ_1_R in primary tumors (*R* ≥ 0.5, *p*-value ≤ 0.05; Supplementary Dataset S4). Notably, there was a striking similarity of the enriched Hallmark pathways (e.g., oxidative phosphorylation, MYC targets) among the genes downregulated in DU145 cells and the genes correlated to σ_1_R in CRPC samples (Fig. 4F). We also found a marked prevalence of mitochondria-related cellular components (e.g., mitochondrial protein complex, mitochondrial inner membrane, mitochondrial matrix, and mitochondrial ribosomal subunits) and biological processes (e.g., mitochondrial translation, mitochondrial gene expression, and respiratory chain complex assembly) among the genes positively correlated to σ_1_R in CRPCs (Fig. 4G, H), highlighting the close relationship between σ_1_R function and mitochondrial homeostasis both in the experimental and clinical settings.

### σ_1_R loss disrupts mitochondrial homeostasis in tumor-initiating stem-like cells

**Fig. 5:**
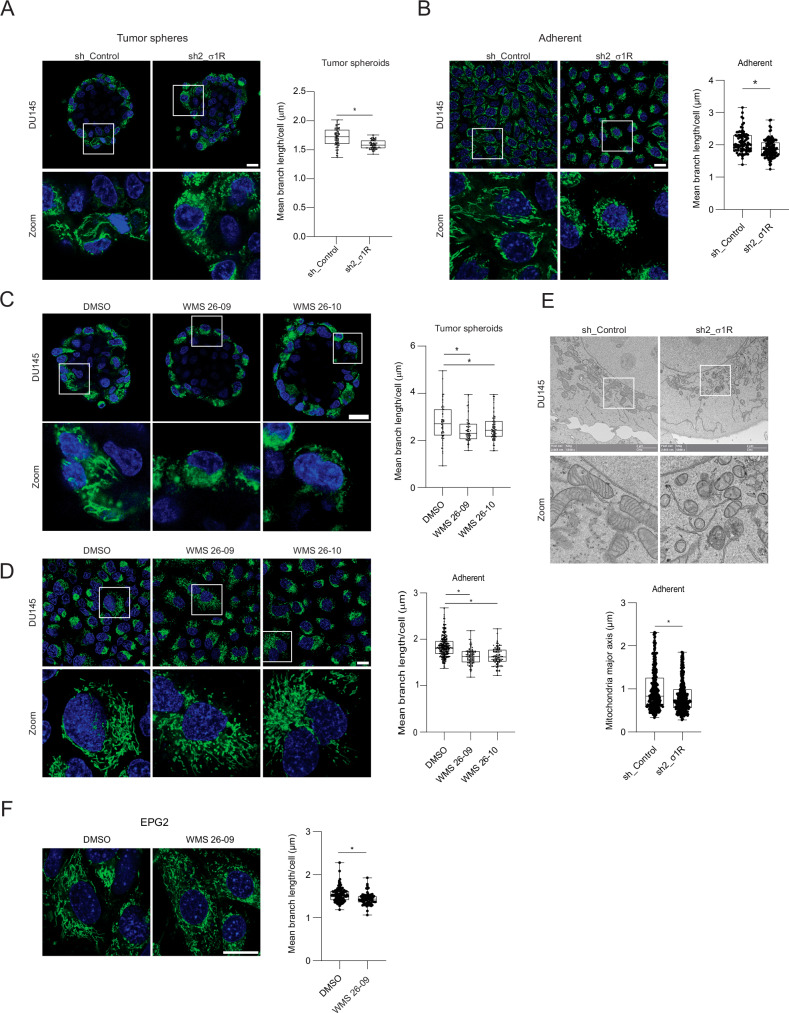
σ_1_R controls mitochondrial dynamics. Mitochondria morphology (MitoTracker Green) and mean branch length in DU145 tumor-sphere (**A**) and bulk adherent cells (**B**) after σ_1_R knockdown. Mitochondria morphology (MitoTracker Green) and mean branch length in DU145 tumor-sphere (**C**) and bulk adherent cells (**D**) treated with σ_1_R antagonists. **E** Transmission electron microscopy (TEM) analysis of mitochondria shape and size in DU145 cells after σ_1_R knockdown. Bar. 2 µm. **F** Mitochondria morphology (MitoTracker Green) and mean branch length in EPG2 cells treated with a σ_1_R antagonist. Bar. 20 µm. Boxplots represent median, interquartile range, maximum and minimum. Data are mean ± SD; *P < 0.01 by *t-*test and ANOVA.

Transcriptomic and proteomic data identified altered mitochondrial homeostasis as a relevant consequence of the loss of σ_1_R function, which could consequently compromise CSC proficiency. σ_1_R was associated predominantly with the ER and mitochondria in DU145 cells (Supplementary Fig. S5A, B), in line with its preferential localization at the MAMs and functional contribution to ER-mitochondria interactions. Specialized protein complexes at the ER-mitochondrial contact points stabilize the inter-organelle interactions and coordinate mitochondrial fusion and fission events [42–47]. Hence, we examined whether σ_1_R inhibition could affect mitochondria in bulk and tumor sphere-forming stem-like cancer cells. Confocal microscopy analysis revealed that bulk adherent tumor cells and stem-like cancer cells accumulated abnormally shortened mitochondria (Fig. 5A, B), indicative of altered mitochondrial fission in response to the σ_1_R depletion [48]. Consistently, the σ_1_R antagonists induced similar mitochondrial morphological changes (Fig. 5C, D). The quantitative analysis of high-resolution confocal microscopy images using mitochondria imaging analysis tools revealed a significant reduction of the mean mitochondrial length (mean branch length) in tumor-sphere and bulk tumor cells upon σ_1_R inhibition (Fig. 5A–D). Transmission electron microscopy (TEM) further confirmed the substantial shortening of mitochondria after σ_1_R knockdown (Fig. 5E). We observed similar morphological changes in the mitochondria of EPG2 cells treated with σ_1_R antagonists (Fig. 5F). Various image analysis tools applied to confocal microscopy images gave consistent estimates with significantly decreased mitochondrial length in σ_1_R-depleted cells (Supplementary Fig. S5C). Other quantitative parameters (i.e., mean of network branches and mean sum of branch length) reflected the increased fragmentation and complexity of the mitochondrial network in the σ_1_R-depleted cells (Supplementary Fig. S5D, E). Instead, the mitochondrial footprint, an estimate of the total extension of the mitochondria network (Supplementary Fig. S5F), and the mitochondrial mass determined by flow cytometry (Supplementary Fig. S5G) did not change after σ_1_R depletion. Notably, despite the impact on the mitochondrial network morphology, σ_1_R depletion did not alter the level of various proteins (i.e., DRP1, MFN2, MFF) directly involved in mitochondrial dynamics control (Supplementary Fig. S5H).

**Fig. 6:**
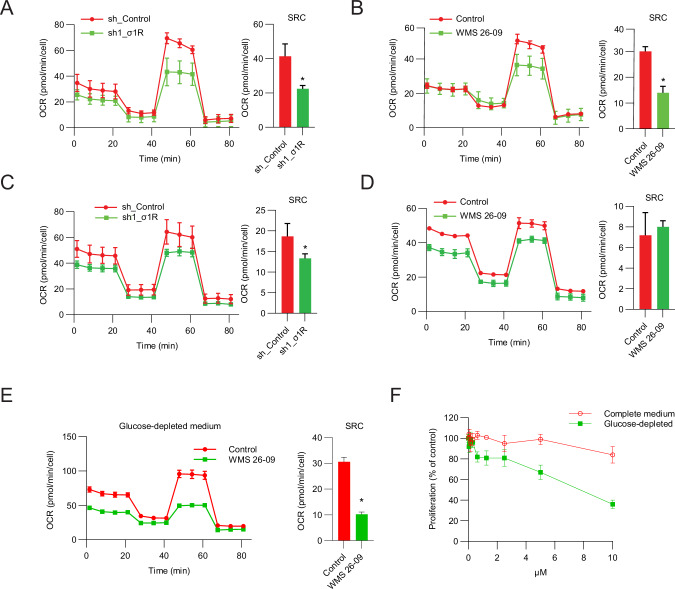
σ_1_R inhibition impairs mitochondrial respiration. **A**, **B** Oxygen consumption rate (OCR) and spare respiratory capacity (SRC) in tumor-sphere cells after σ_1_R knockdown (**A**) and σ_1_R antagonist (10 µM) treatment for 24 h (**B**). OCR and SRC in DU145 cells grown in adherent conditions after σ_1_R knockdown (**C**) and σ_1_R antagonist treatment for 24 h (**D**). **E** Oxygen consumption rate (OCR) and spare respiratory capacity (SRC) in DU145 cells grown in adherent conditions and treated with σ_1_R antagonist for 24 h in glucose-depleted medium. **F** Proliferation of DU145 cells grown in adherent conditions and treated with σ_1_R antagonist in complete or glucose-depleted medium. Data are mean ± SD; * *P* < 0.01 by *t-*test.

Concurrent with the altered morphology, measurement with the Seahorse flux analyzer showed reduced mitochondrial function with impaired mitochondrial spare respiratory capacity (SRC) in tumor sphere-forming stem-like cells after σ_1_R knockdown (Fig. 6A) and treatment with σ_1_R antagonists (Fig. 6B). Thus, σ_1_R inhibition induced a marked accumulation of abnormal and dysfunctional mitochondria in CSCs. Conversely, mitochondrial function in bulk tumor cells was not or minimally affected by genetic depletion and treatment with σ_1_R antagonists (Fig. 6C, D). Thus, σ_1_R inhibition caused different consequences in specific cell contexts. Inhibition of σ_1_R disrupted mitochondrial homeostasis and function in tumor sphere-forming stem-like cancer cells and likely was incompatible with their self-renewal and continuous expansion. Instead, bulk tumor cells apparently could cope better with the consequences of σ_1_R inhibition without a significant impact on mitochondrial function and homeostasis under physiological conditions. Interestingly, when challenged by glucose starvation, bulk tumor cells became more susceptible to σ_1_R antagonists, exhibiting higher mitochondrial dysfunction (Fig. 6E) and growth inhibition (Fig. 6F).

### σ_1_R links mitochondrial homeostasis and Wnt/β-catenin signaling to CSC proficiency

**Fig. 7:**
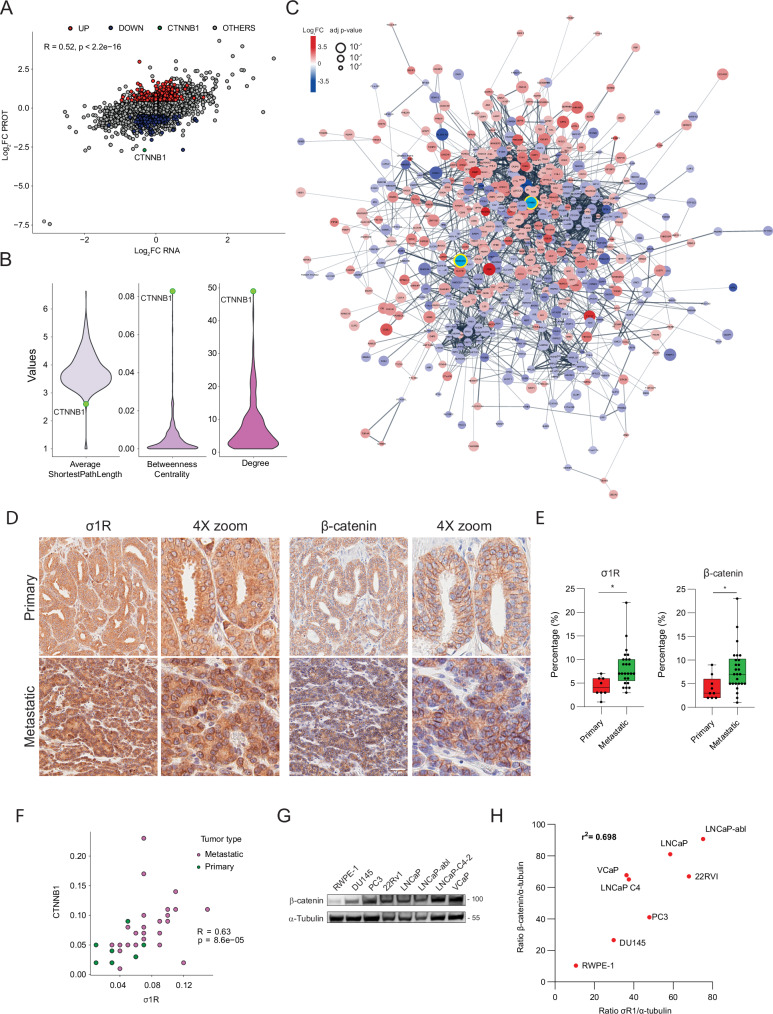
σ1R enhances β-catenin signaling. **A** Differential expression (log_2_FC) of proteins (PROT) and transcripts (RNA) in σ_1_R-depleted cells. **B** Local network parameters with the interconnectivity levels among the proteins differentially expressed after σ_1_R knockdown. **C** Network interactions among the differentially expressed proteins (|log2FC|>0.7) in σ_1_R-depleted cells. σ_1_R and β-catenin are circled in yellow. **D** Immunodetection of σ_1_R and β-catenin proteins in human primary and metastatic prostate tumors. Bar, 50 µm. **E** Percentage of σ_1_R and β-catenin positive cells in human primary and metastatic prostate cancers determined by immunohistochemistry. **F** Spearman correlation analysis of σ_1_R and β-catenin protein expression in human primary and metastatic prostate cancers. **G** β-Catenin protein expression levels examined by Western blotting. **H** Correlation analysis of σ_1_R and β-catenin protein levels in human prostate cell lines. Boxplots represent median, interquartile range, maximum and minimum. Data are mean ± SD; *P < 0.01 by *t-*test.

We found that σ_1_R inhibition had a crucial impact on CSC self-renewal. Disrupting mitochondrial dynamics σ_1_R inhibition could interfere with mitochondrial partitioning in the CSC progeny, impairing asymmetric stem cell division and unlimited expansion. The σ_1_R inhibition and the disruption of mitochondrial homeostasis might generate additional negative signals contributing to the arrest of the expansion of stem-like cancer cells. Hence, we further examined the transcriptomic and proteomic data from σ_1_R-depleted cells to search for additional targets of the σ_1_R loss. Notably, integrating proteomic and transcriptomic data revealed a group of proteins significantly downregulated in σ_1_R-depleted cells but not affected at the transcriptional level, hinting at a post-transcriptional mechanism (Fig. 7A). β-Catenin (CTNNB1) was the top-ranking downregulated protein within this group (Supplementary Dataset S5). A protein network analysis showed that β-catenin was a top-interacting protein and primary hub among the proteins affected by σ_1_R loss (Fig. 7B, C and Supplementary Dataset S6), suggesting that it could be a critical player mediating the effects of σ_1_R in prostate cancer cells.

β-Catenin is a central component of the Wnt/β-catenin pathway, a canonical developmental and stem cell signaling pathway [49]. Wnt signaling activation is associated with enhanced stemness, disease progression, and treatment resistance in prostate cancer [49, 50]. Hence, a functional link between σ_1_R and β-catenin could be highly relevant clinically. By controlling β-catenin, σ_1_R could sustain Wnt signaling and cancer stemness in prostate tumors. In support of a relation between β-catenin and σ_1_R function, we found consistent co-expression and co-localization of the two proteins in clinical samples of primary and metastatic prostate cancers (Fig. 7D). σ_1_R and β-catenin were significantly upregulated in metastatic compared to primary tumors (Fig. 7E) and were highly correlated in tumor samples (Fig. 7F). Interestingly, we also found a positive correlation between σ_1_R and β-catenin protein levels in prostate cancer cell lines (Fig. 7G, H).

**Fig. 8:**
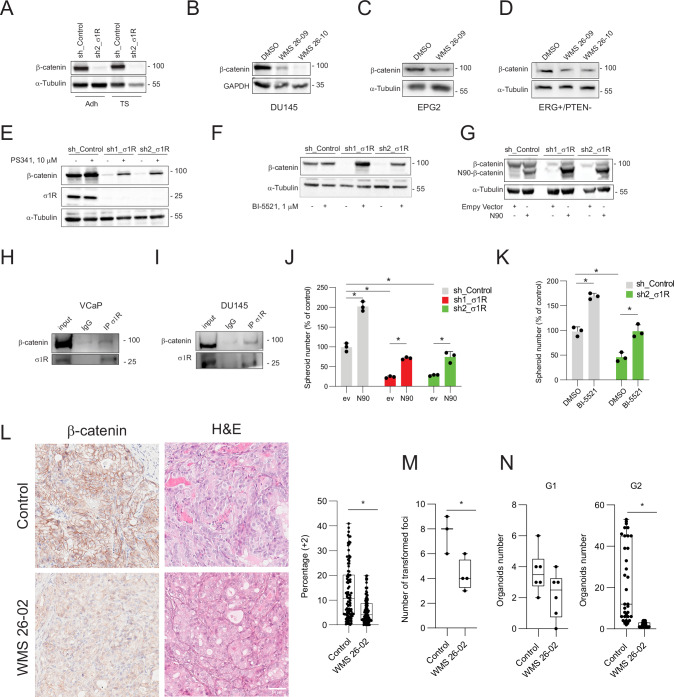
σ_1_R links β-catenin stability and CSC proficiency. **A** β-Catenin expression in bulk adherent (Adh) and tumor-sphere (TS) cultures of control (sh_Control) and σ_1_R-depleted DU145 cells. **B–D** β-Catenin protein levels in DU145 (**E**), EPG2 (**F**), and ERG^+^/PTEN^-^ derived (**G**) cells after treatment with σ_1_R antagonists (10 µM, 24 h). **E** β-Catenin protein in control and σ_1_R-depleted DU145 cells after treatment with PS341 (10 µM, 24 h). **F** β-Catenin protein in control and σ_1_R-depleted DU145 cells after treatment with BI-5521 (1 µM, 24 h). **G** Endogenous wild-type and mutant (N90) β-catenin protein in control and σ_1_R-depleted DU145 cells. **H**, **I** Co-immunoprecipitation of σ_1_R and β-catenin in VCaP (**H**) and DU145 (**I**) cells. **J** Tumor-sphere formation after expression of mutant (N90) β-catenin or empty vector (ev) in control and σ_1_R-depleted DU145 cells. **K** Tumor-sphere formation after treatment of control and σ_1_R-depleted DU145 cells with BI-5521 (1 µM, 24 h). **L** Immunodetection of β-catenin and HE stains in ERG^+^/PTEN^-^ prostate tumors after treatment with vehicle and WMS 26-02 (40 mg/Kg). Bar. 50 µm. Right panel, Percentage of β-catenin-positive cells in mouse prostates from control and WMS 26-0- treated mice. **M** Frequency of invasive foci in prostates of control and WMS 26-02 treated ERG^+^/PTEN^−^ mice. **N** Ex vivo organoid formation at consecutive generations (G1 and G2) by tumor cells isolated from mouse prostates of control and WMS 26-02 treated ERG^+^/PTEN^−^ mice. Boxplots represent median, interquartile range, maximum and minimum. Data are mean ± SD; * *P* < 0.01 by *t-*test and ANOVA.

Supported by these findings, we investigated further the functional relationship between σ_1_R and β-catenin in our experimental models. Genetic knockdown of σ_1_R reduced the β-catenin protein level in bulk and tumor-sphere cells (Fig. 8A). Treatment with σ_1_R antagonists similarly reduced β-catenin in DU145 and EPG2 cells and ERG^+^/PTEN^-^-derived tumor spheres (Fig. 8B–D). In support of a direct effect on β-catenin protein turnover [49], blocking the proteasome (Fig. 8E) or GSK-3β-mediated phosphorylation (Fig. 8F) prevented the loss of β-catenin caused by σ_1_R knockdown. The β-catenin mutant (N90) lacking the N-terminal domain was also resistant to degradation and was not affected by σ_1_R depletion (Fig. 8G). On the other hand, co-immunoprecipitation experiments showed that σ_1_R and β-catenin interacted in VCaP and DU145 cells (Fig. 8H, I), suggesting that, despite the low amount of σ_1_R-bound β-catenin, the physical interaction with σ_1_R could in part contribute to β-catenin protein stability. Furthermore, restoring the β-catenin protein level by expressing the constitutively active N90 mutant (Fig. 8J) or inhibiting GSK-3β (Fig. 8K) rescued tumor sphere formation in σ_1_R-depleted cells, demonstrating that β-catenin degradation contributed substantially to the loss of CSCs caused by σ_1_R inhibition. The GSK-3β inhibitor also restored mitochondrial morphology in σ_1_R-depleted bulk and tumor sphere-forming cells (Supplementary Fig. S6A, B), in line with reciprocal crosstalks between the Wnt pathway and mitochondrial biogenesis [51, 52]. Consistently, β-catenin knockdown partially reduced tumor sphere formation and cell proliferation, reproducing the phenotypic changes induced by σ_1_R inhibition (Supplementary Fig. S6C, D).

Our data indicate that the combined effect of σ_1_R inhibition on mitochondria homeostasis and Wnt/β-catenin signaling led to impaired CSC self-renewal and induced progressive exhaustion of their proliferative potential. To test this concept in an in vivo model, we treated ERG^+^/PTEN^-^ mice with the σ_1_R antagonist WMS 26-02 and assessed the mouse prostates at the end of the treatment. We observed a substantial reduction of β-catenin protein level in WMS 26-02-treated tumors compared to the control group (Fig. 8L). In contrast, other markers, like AR and ERG, were not affected (Supplementary Fig. S7A). We also found a significant decrease in invasive areas in WMS 26-02-treated prostate tumors (Fig. 8M and Supplementary Fig. S7B). Furthermore, prostate tumor cells dissociated from WMS 26-02-treated tumors exhibited a reduced ability to form organoids at consecutive generations in ex vivo assays (Fig. 8N), confirming the functional impairment of tumor-initiating stem-like cells in response to σ_1_R inhibition.

## Discussion

Mitochondrial homeostasis and mitochondrial-cell communications are essential for balancing the differentiation and self-renewal potential of CSCs [9, 10]. Here, we show that σ_1_R has a cardinal role in integrating mitochondrial dynamics and mitochondrial-nuclear signaling and determining the fate of the CSC progeny. σ_1_R is a ligand-operated chaperone implicated in multiple intracellular signaling pathways. We found that σ_1_R ensures efficient mitochondrial division and signaling through the Wnt/β-catenin pathway in tumor-initiating stem-like cells, promoting their self-renewal and continuous expansion. Inhibition of σ_1_R profoundly affected the self-renewal and tumorigenic potential of CSCs, thus establishing σ_1_R as an actionable target for developing effective CSC-directed anticancer therapies.

σ_1_R is an integral ER membrane protein located primarily at the MAM. σ_1_R functions as a ligand-operated chaperone and interacts with components of the ER, mitochondria, and other organelles, modulating protein stability and activity in a cell-type- and context-specific manner [18]. Through multiple protein-protein interactions, σ_1_R can operate as a modulator of inter-organelle signaling [18, 19], including communications between the ER, mitochondria, and the nucleus [18, 19]. At the level of mitochondria, we show that inhibiting σ_1_R induces changes reminiscent of uncoordinated fission with the accumulation of shortened, distorted, and dysfunctional organelles and an abnormal mitochondrial network. These changes likely reflect the disruption of ER-mitochondria contacts and altered interactions with proteins that regulate mitochondrial dynamics and network organization [42, 46, 47]. σ_1_R interacts with MNF2 and increases MFN2-dependent ER-mitochondria contacts, leading to abnormally enlarged mitochondria in adipocytes [46]. σ_1_R also interferes with mitochondrial fission proteins, like Fis1 and Drp1, modulating the availability and activity of multiple factors that regulate the dynamic changes in mitochondrial shape, motility, and function [48, 53, 54].

The accumulation of abnormal and dysfunctional mitochondria caused by σ_1_R inhibition had dramatic effects on the CSC population. CSCs rely on mitochondrial dynamics and motility for mitochondria segregation and quality control during asymmetric stem cell division, a critical step for ensuring the propagation of proficient CSC progeny [9, 11]. We used tumor sphere-forming assays to identify cancer stem-like cells functionally and assess their response to σ_1_R inhibition. This method overcomes the challenges of other approaches based on pre-defined sets of cell surface markers and sorting of the putative CSC population, which may be challenging to apply reproducibly to diverse sources and tumor subtypes [8, 30, 41]. We showed that interfering with σ_1_R impaired the self-renewal capability and inhibited the expansion of stem-like cancer cells in 3D tumor sphere cultures. Indeed, the self-renewal and propagation of CSCs for subsequent generations as tumor spheres were impaired persistently both in vitro and ex vivo. Bioinformatics analysis of RNA-seq data showed the downregulation of stem cell marker genes and confirmed the impact of σ_1_R knockdown on the stem cell-like phenotype. Interestingly, reflecting the complexity of prostate cancer progression with aberrant epithelial-mesenchymal plasticity [55], we found typical epithelial genes (e.g., CHD1), which are upregulated along with stem cell marker genes in prostate cancer cell lines and metastatic tumors cancer [41, 56–59], significantly downregulated by σ_1_R knockdown in DU145 cells. Bulk tumor cells tolerate better the σ_1_R inhibition and the consequent mitochondrial dysfunction and continue to proliferate in 2D cultures. Compensatory mechanisms likely allow the gradual elimination of the dysfunctional mitochondria and dilute the impact of inhibiting σ_1_R on the overall population of bulk tumor cells without affecting their survival and proliferation. Instead, the segregation and elimination of dysfunctional mitochondria is critical for stem-like cancer cells. Partitioning of functional mitochondria to dividing CSCs is essential for maintaining the progeny of daughter cells with intact stem cell properties and self-propagating and tumorigenic capability. Indeed, the inheritance of fully functional mitochondria at the time of cell division defines the quality of the CSC progeny and their performance in terms of tumorigenic capability [10, 11]. Hence, the incorrect segregation of functional and dysfunctional mitochondria compromises the CSC progeny, leading to the progressive loss of their self-replicative and tumorigenic potential. Accordingly, we observed drastically reduced self-renewal and regrowth of stem-like cancer cells in G2 tumor sphere assays after exposure to σ_1_R antagonists. Furthermore, the genetic knockdown of σ_1_R reduced the expansion and tumorigenic proficiency of the tumor-initiating stem-like cells and prevented tumor growth in mice. Treatment of transgenic mice with a σ_1_R antagonist provided further evidence of the impact of σ_1_R inhibition in vivo, demonstrating the inhibition of the Wnt/β-catenin stem cell signaling and reduced tumor-initiating capability in an orthotopic prostate cancer model.

Various developmental and stem cell pathways sustain CSC proliferation and self-renewal [60]. We show here that σ_1_R contributes to the Wnt/β-catenin signaling by protecting β-catenin from proteasomal degradation. Consistently, genetic knockdown of β-catenin phenocopied the effects of σ_1_R inhibition in tumor-sphere assays, whereas blocking β-catenin degradation prevented the impact of inhibiting σ_1_R on CSCs. Despite the amount of σ_1_R-bound β-catenin in whole cell immuno-precipitates is a minimal fraction of the total β-catenin, the direct interaction between β-catenin and σ_1_R could contribute significantly to the β-catenin protein stability. Indeed, we cannot exclude that the σ_1_R-bound β-catenin is a fraction of the cellular β-catenin that resides in a subcellular compartment (e.g., ER) functionally relevant for its biosynthesis, intracellular distribution, and turnover [61–63]. On the other hand, the massive disruption of mitochondria homeostasis caused by σ_1_R loss indirectly could act as a trigger for Wnt/β-catenin signaling inactivation and β-catenin degradation, thus contributing to the effects of σ_1_R inhibition [52, 63]. Ultimately, through this dual control of mitochondrial homeostasis and β-catenin stability, σ_1_R activation can promote the uncontrolled expansion of tumor-initiating stem-like cells.

This study reveals relevant crosstalk between σ_1_R, mitochondria, and β-catenin in prostate cancer models. The Wnt/β-catenin pathway is associated with castration resistance and the emergence of aggressive CRPC subtypes [64, 65]. Consistently examining clinical samples of primary and metastatic prostate cancers, we found a tight correlation between σ_1_R and β-catenin protein levels and significantly increased expression of both σ_1_R and β-catenin in metastatic CRPCs. On the other hand, transcriptomic data showed a tight association of σ_1_R with mitochondria-related processes, preferentially in CRPC patients. Thus, σ_1_R activation connects to both enhanced mitochondrial dynamics and β-catenin stability in advanced prostate cancers. The control of mitochondrial homeostasis and Wnt/β-catenin signaling by σ_1_R, therefore, could synergistically contribute to the progression to metastatic and castration-resistant tumors. There is also evidence that Wnt/β-catenin signaling and mitochondrial homeostasis can reciprocally influence each other. Mitochondria signaling can modulate the expression of components of the Wnt/β-catenin pathway [52, 66], and the Wnt/β-catenin signaling can influence the transcription of genes implicated in mitochondrial biogenesis and function [51, 67, 68]. Hence, the reduced Wnt/β-catenin signaling upon loss of σ_1_R function could further delay any attempt to recover mitochondrial function and contribute to the persistent impairment of CSC capacity.

In conclusion, our data indicate that σ_1_R constitutes a relevant signaling hub between multiple intracellular compartments, connecting mitochondria homeostasis and Wnt/β-catenin signaling and having a profound impact on the ability of CSCs to proliferate and self-sustain. σ_1_R controls mitochondrial dynamics to ensure the timely and proper partitioning of functional mitochondria in the daughter CSCs. σ_1_R also directly influences β-catenin protein turnover and signaling through the Wnt/β-catenin pathway to promote the proliferation of the CSC progeny. Thus, σ_1_R integrates multiple signals essential for coupling mitochondria and CSC division. We propose that σ_1_R preserves the high replicative and self-renewal capability of CSCs by coordinating the cell division and mitochondrial segregation among the daughter CSCs. Hence, inhibition of σ_1_R profoundly affects the CSC progeny, causing the progressive exhaustion of the self-renewal capability and loss of tumorigenic potential. Importantly, by driving the expansion and self-renewal of prostate CSCs, σ_1_R could be instrumental in promoting tumor regrowth and recurrence after treatment with AR pathway inhibitors, cytotoxic drugs, and immunotherapeutics in prostate cancer patients. Indeed, we are investigating the feasibility and safety of combinations of σ_1_R antagonists with currently standard therapies for metastatic prostate cancer to see if they improve treatment efficacy in preclinical trials. Because of its central role in CSC biology, the σ_1_R-centered signaling axis uncovered here is a promising target for developing selective CSC-directed therapies and novel strategies for cancer treatment.

## Supplementary information

Supplementary information file_ Supplementary figures, Extended Methods and Reagents list

Dataset S1

Dataset S2

Dataset S3

Dataset S4

Dataset S5

Dataset S6

## Data Availability

Data and materials that support the findings of this study are available within the article and supplemental information. Supplemental figures and datasets are available as supplemental information. RNA-sequencing data reported in this study are deposited in the NCBI Gene Expression Omnibus (GEO, GSE203198). Any additional information required to reanalyze the data reported in this paper is available from the lead contact upon request.
