# Supplementary material for: Integrated control of cancer stemness by σ1 receptor in advanced prostate cancer
Source: Oncogene. 2025 Sep 2;44(42):4032–46. doi: 10.1038/s41388-025-03541-7 (PMC12518131; doi:10.1038/s41388-025-03541-7)
Supplement: Supplementary file 1 — Supplementary information file_ Supplementary figures, Extended Methods and Reagents list [file 41388_2025_3541_MOESM1_ESM.pdf]

SUPPLEMENTARY INFORMATION FILE

Integrated control of mitochondrial homeostasis and cancer stemness by σ_1 receptor in advanced prostate cancer

Gianluca Civenni,[1] Giada Sandrini,[1,2] Jessica Merulla,[1] Carola Musumeci,[1] Elisa Federici,[1] Arianna Vallegra,[3] Aleksandra Kokanovic,[1] Simone Mosole,[1] Dheeraj Shinde,[1] Elisa Sorrenti,[1] Alyssa J. J. Paganoni,[1] Martina Marchetti,[1] Riccardo Valzelli,[1] Domenico Albino,[1] Matteo Pecoraro,[4] Andrea Rinaldi,[1] Marco Bolis,[1,2,3] Roger Geiger,[1,4] Tobias Winge,[5] Catharina Holtschulte,[5] Erik Laurini,[6] Sabrina Pricl,[6] Giuseppina M. Carbone,[1] Bernhard Wünsch,[5] and Carlo V. Catapano[1,*]

The supplementary information file includes:

- Supplementary Figures
- Detailed Materials and Methods
- Reagents and Resources Table
- Supplementary Dataset Files

Figure S1

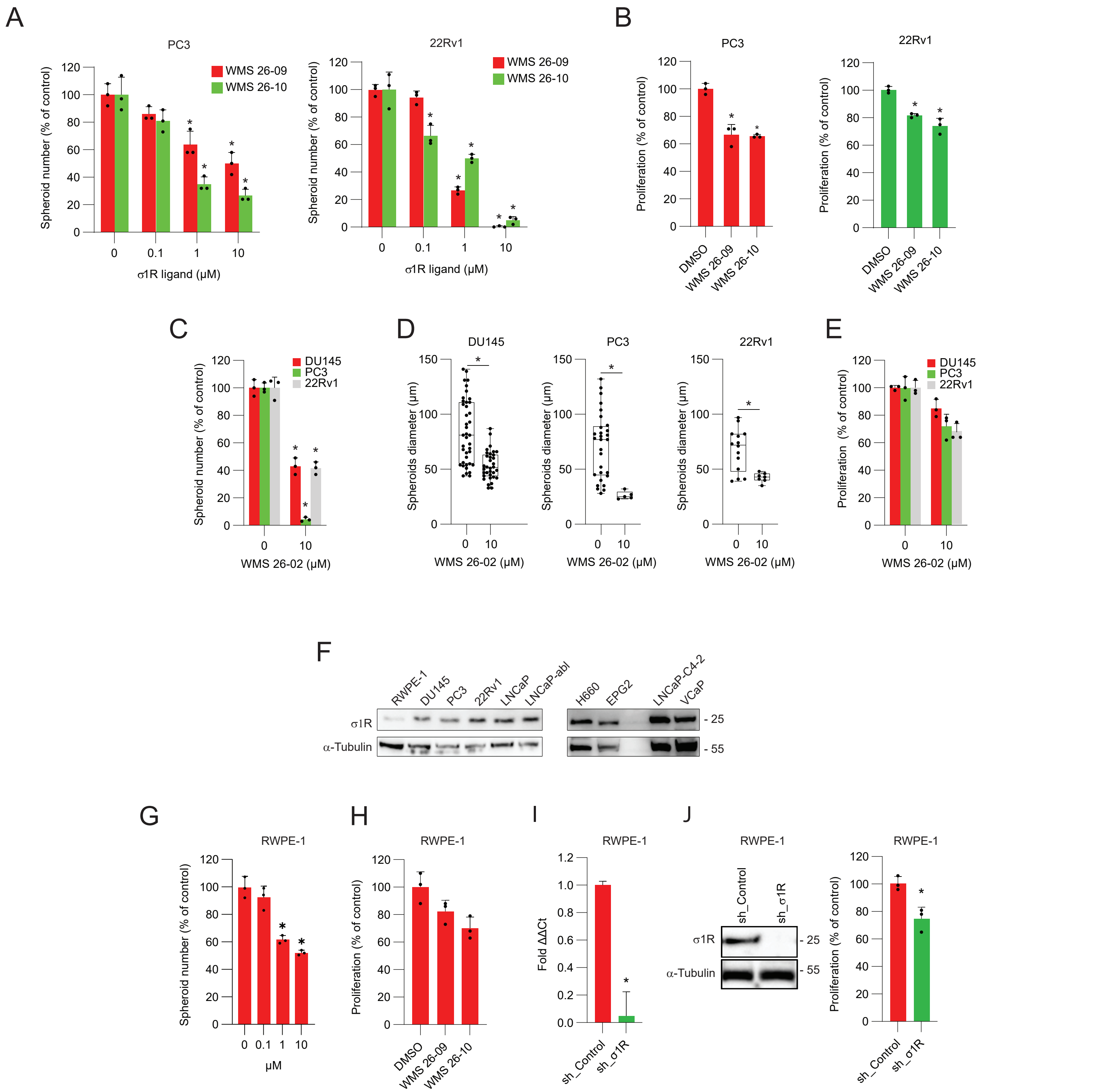

Figure S1. σ1R expression and sensitivity of prostate cells to receptor antagonists. A. Tumor-spheres of PC3 and 22Rv1 cells after treatment with WMS 26-09 and WMS 26-10. **B.** Proliferation of PC3 and 22Rv1 cells after treatment with WMS 26-09 and WMS 26-09 (10 μM). **C-D.** Number (C) and size (D) of tumor-spheres of DU145, PC3, and 22Rv1 cells incubated with WMS 26-02. **E.** Proliferation of DU145, PC3, and 22Rv1 cells incubated with WMS 26-02 (10 μM, 72 h). **F.** σ1R protein expression in human and murine prostatic cell lines. **G.** Prostate-sphere numbers of RWPE-1 cells after treatment with WMS 26-09. **H.** Proliferation of RWPE-1 cells treated with σ1R antagonists (10 μM, 72 h). **I-J.** Knockdown of σ1R and impact on proliferation in RWPE cells. Boxplots represent median, interquartile range, maximum and minimum. Data are mean ± SD * P < 0.01 by t-test and ANOVA.

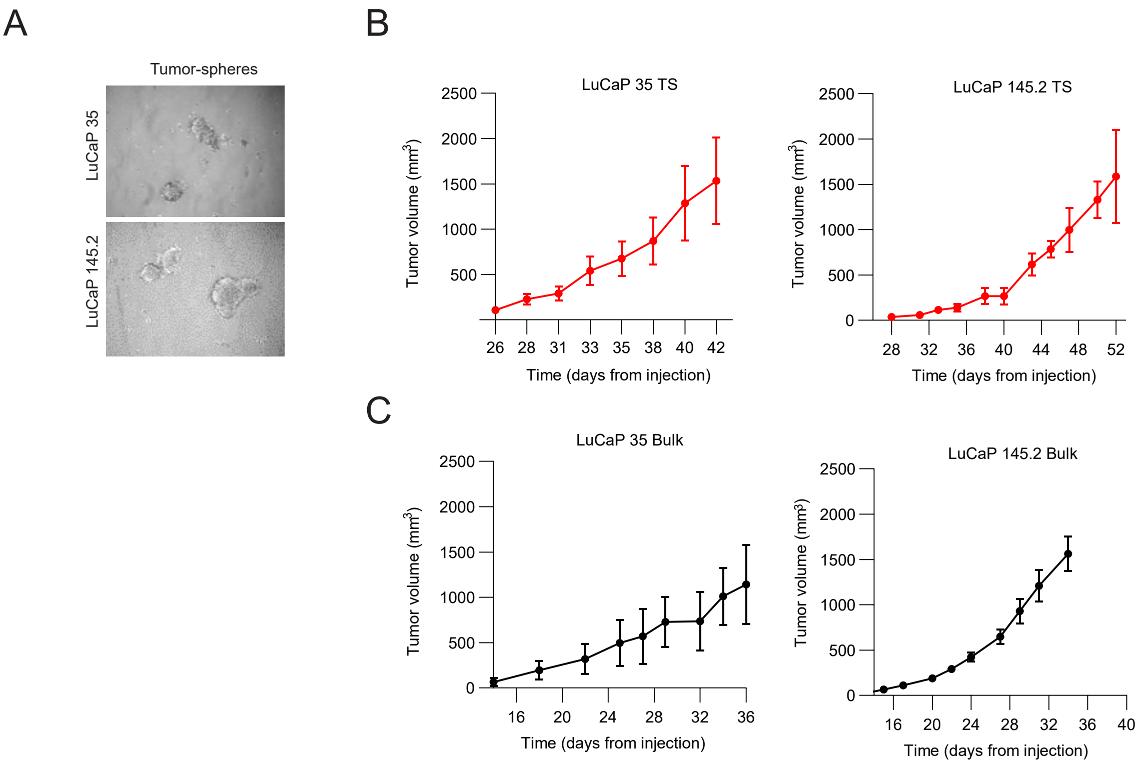

Figure S2. In vitro growth and in vivo tumorigenicity of tumor sphere-forming stem-like cancer cells from patient-derived xenografts. A. Representative image of tumor-sphere generated by LuCaP 35 and LuCaP 145.2 cells. **B.** Growth of tumor-sphere-forming cells (10^4 cells/mouse) derived from LuCaP 35 and LuCaP 145.2 PDXs and implanted subcutaneously in NSG mice. **C.** Growth of bulk tumor cells (10^6 cells/mouse) derived from LuCaP 35 and LuCaP 145.2 PDXs and implanted subcutaneously in NSG mice.

Figure S3

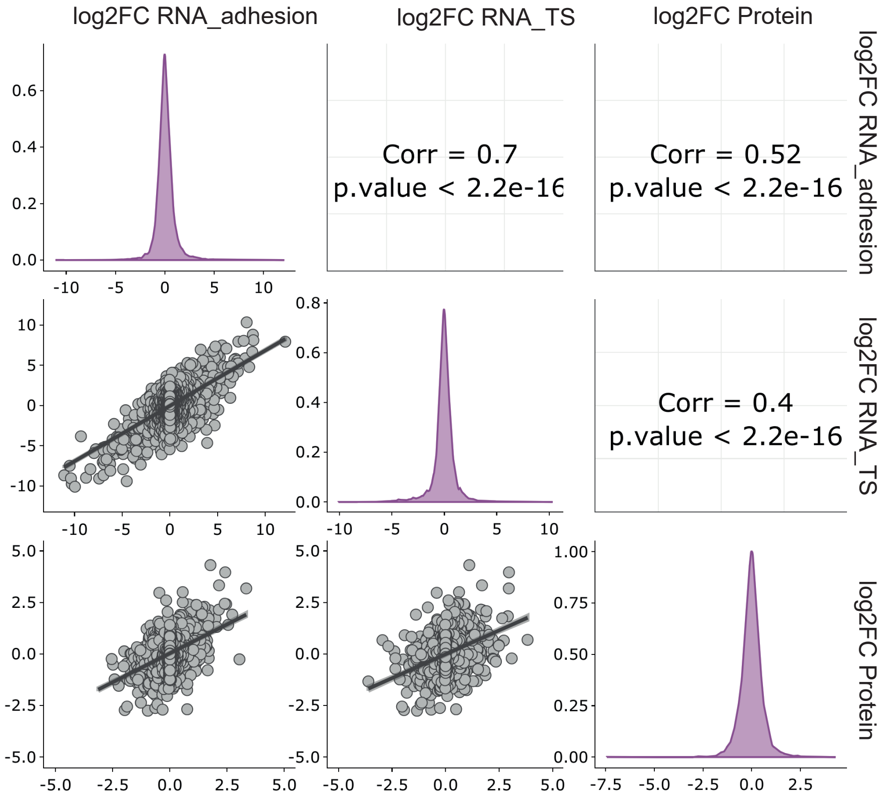

Figure S3. Analysis of transcriptomic and proteomic data in control and σ1R-depleted DU145 cells. Correlation between the log2FC of the shRNA vs. shControl comparisons in the transcriptomic and proteomic data in the diverse experimental settings. Pearson correlation indexes and the p-values (top right panels), scatter plots with linear regression (bottom left panels), and density profiles of the single experiments (diagonal plots) are represented.

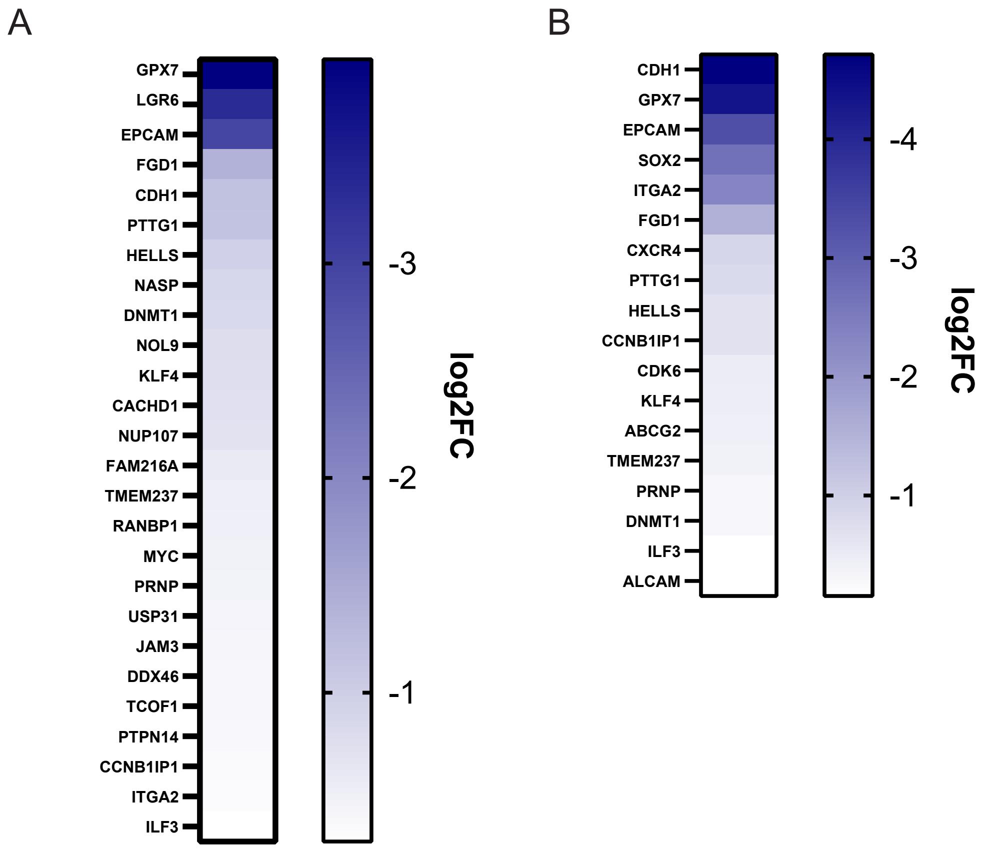

Figure S4. Stem cell marker genes significantly down-regulated in DU145 cells after σ1R knockdown. Heatmap shows the log2 Fold change (log2FC) of the genes modulated significantly ($P < 0.05$) in bulk (A) and stem-like (B) DU145 cells.

Figure S5

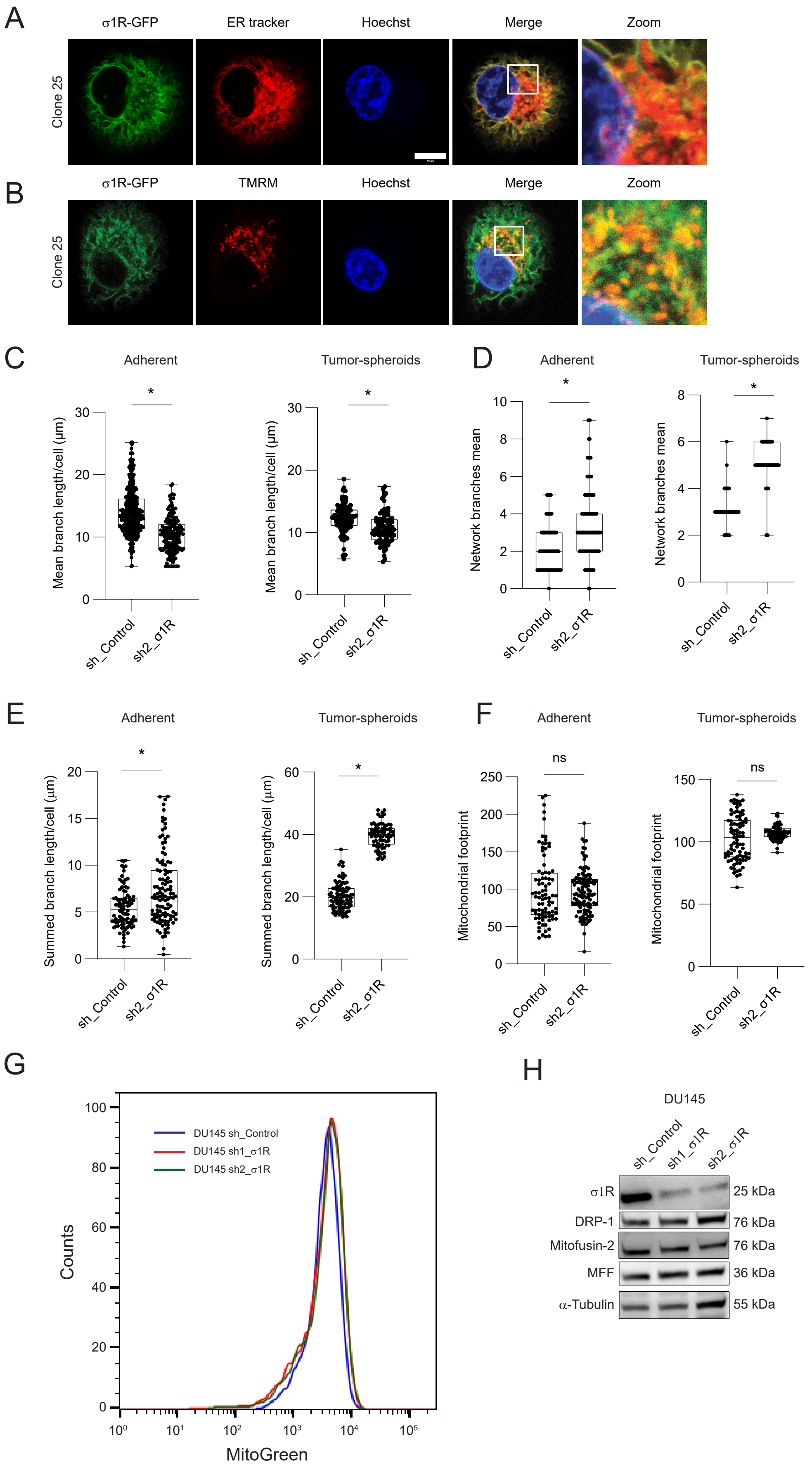

Figure S5. Intracellular localization and impact of σ1R inhibition in DU145 cells. A-B. Localization of GFP-tagged σ1R, endoplasmic reticulum (A, ER tracker), and mitochondria (B, TMRM) in DU145 cells (clone 25). Nuclei are stained with Hoechst. Images with single and merged channels are shown. Bar: 10 µm. **C.** Mitochondrial mean branch length in adherent and tumor-sphere forming DU145 cells with σ1R knockdown determined with the MitoHacker. **D-F.** Mitochondrial network parameters, mean network branches (D), summed branch length (E), and mitochondrial footprint (F) determined by MiNA in adherent and tumor-spheres forming DU145 cells with and without σ1R knockdown. **G.** Flow cytometry analysis of the total mitochondria stained with MitoTracker Green in control and σ1R-depleted DU145 cells. **H.** Expression of DRP-1, MFN2, and MFF in control (sh_Control) and σ1R-depleted (sh1_σ1R, sh2_σ1R) DU145 cells. Boxplots represent median, interquartile range, maximum and minimum. Data are mean ± SD * P < 0.01 by t-test.

Figure S6

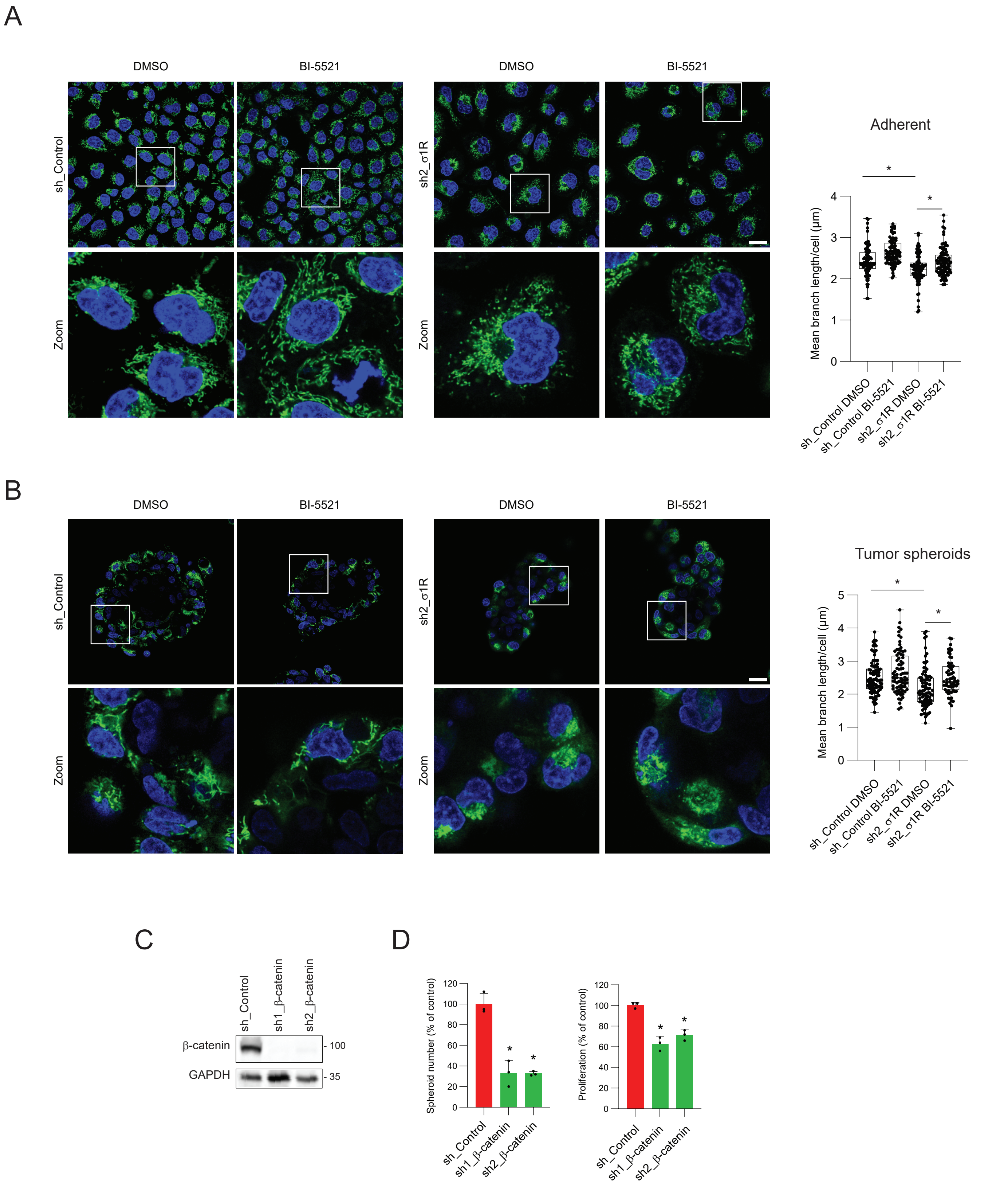

Figure S6. Mitochondria morphology in DU145 cells. A-B. Mitochondria morphology and mean branch length in bulk adherent (A) and tumor sphere (B) cultures of DU145 cells after σ1R knockdown and treatment with BI5512 (1 μM, 24 h). Mitochondria were stained with MitoTracker Green for 40 min. Nuclei were stained with Hoechst. Bar: 20 μm. Images with merged channels are shown. **C.** β-Catenin knockdown by shRNA-mediated targeting in DU145 cells. **D.** Tumor sphere growth and cell proliferation in control and β-catenin-depleted DU145 cells. Boxplots represent median, interquartile range, maximum and minimum. Data are mean ± SD; * P < 0.01 by t-test and ANOVA.

Figure S7

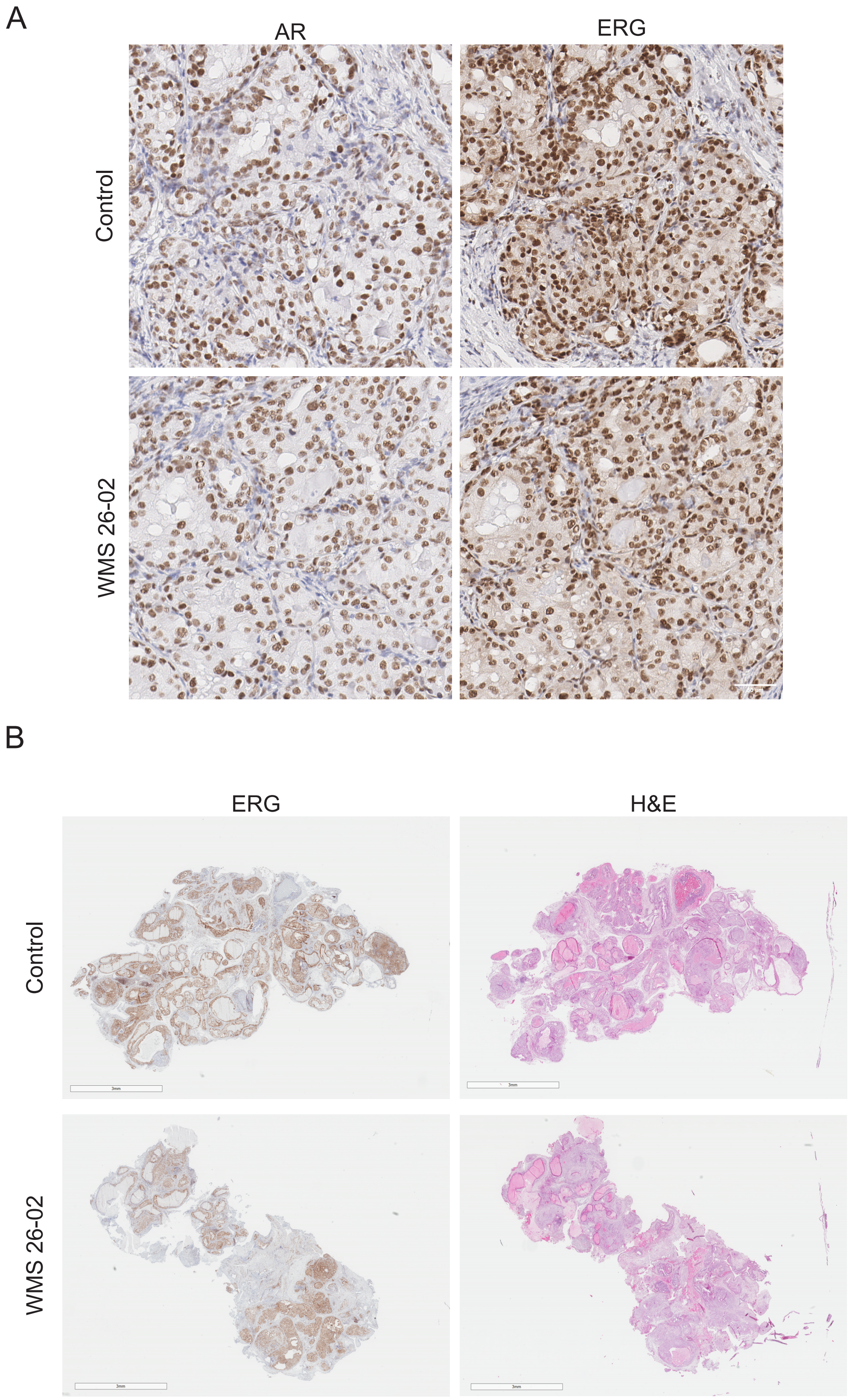

Figure S7. Histopathological and immunohistochemical evaluation of ERG+/PTEN- mouse prostatic tissue. A. Immunodetection of AR and ERG stains in ERG+/PTEN- prostate tumors after treatment with vehicle and WMS 26-02 (40 mg/Kg). Bar: 50 µm. **B.** Histopathology evaluation of tumoral lesions in the prostate of ERG+/PTEN- mice treated with vehicle or WMS 26-02. Tissue sections were stained with H&E and an antibody to human ERG to identify tumor-positive areas. Bar: 3 mm.

DETAILED MATERIALS AND METHODS

Cell lines

Cell lines (DU145, PC3, 22RV1, LNCaP, LNCaP-C4-2, NCI-H660, VCaP, and RWPE-1) were obtained from the American Type Culture Collection (ATCC). DU145, PC3, 22Rv1, and LNCaP cells were maintained in RPMI 1640 medium (21875-034, Gibco®) supplemented with L-Glutamine 2%, 10% fetal bovine serum (FBS) (S1810-500, Biowest), and 1% penicillin-streptomycin (15140-122, Gibco®). VCaP cells were cultured in DMEM medium (61965-026, Gibco®) supplemented with 10% fetal bovine serum (FBS) (S1810-500, Biowest) and 1% penicillin-streptomycin. NCI-H660 cells were maintained in suspension in RPMI 1640 medium (21875-034, Gibco®) supplemented with 0.005 mg/mL Insulin, 0.01 mg/mL Transferrin, 30 nM Sodium selenite, 10 nM Hydrocortisone, 10 nM β-estradiol, 2 mM L-glutamine, 5% fetal bovine serum, and 1% penicillin-streptomycin. LNCaP C4-2 cells were cultured in DMEM medium (61965026, Gibco®)/Ham's F12 Nutrient Mix (11765-054, Gibco®) 4:1 supplemented with 10% fetal bovine serum (FBS), 100 ng/mL Insulin from bovine pancreas (I6634, Sigma-Aldrich), 275 ng/mL 3,3′,5-Triiodo-L-thyronine sodium salt (T6397, Sigma-Aldrich), 88.6 ng/mL apo-Transferrin human (T1147, Sigma-Aldrich), 4.9 ng/mL d-Biotin (ICN19463401, MP Biomedicals™), 251.8 ng/mL Adenine (A2786, Sigma-Aldrich). LNCaP-abl cells (Cellosaurus CVCL_4793) were maintained in phenol red-free RPMI 1640 medium (11835-063, Gibco) supplemented with 10% charcoal-stripped serum (CSS) (12676-029, Gibco) and 1% penicillin-streptomycin (15140-122, Gibco). Human prostate epithelial RWPE-1 cells were maintained in keratinocyte serum-free growth medium (17005042, Gibco®) supplemented with human recombinant Epidermal Growth Factor (rEGF) and Bovine Pituitary Extract (BPE). Cells were regularly checked for *Mycoplasma* contamination using MycoAlert Mycoplasma detection kit (Lonza).

Stable cell lines expressing σ1R-GPF tagged were produced by transfection of the expression vectors using JetPrime® (Thermo Fisher) and selection with puromycin (0.5 µg/mL; Sigma-Aldrich). Vectors for expression of σ1R (GFP-σ1R, RG201206) were obtained from Origene (www.origene.org). Expression of the ectopic protein was monitored by flow cytometry. Cell lines with stable knockdown of σ1R were produced by infection with Ready-to-transduce lentiviral particles expressing shRNAs (clone ID: NM_005866.2-657s21c1) and selection with puromycin. Transient knockdown of σ1R was obtained by transfection of siRNA (100 nM with Lipofectamine 2000, Invitrogen). siRNA directed to σ1R (Thermo Fischer, 111615) and control siRNA (siGL3) were purchased from Ambion. Murine EPG2 cells were established from prostate tumors generated in $ERG^{+}/PTEN^{-}$ (*Pb-Cre4;Pten*$^{flox/flox}$*;R26*^{ERG}) transgenic mice. Detailed procedures will be described elsewhere. Briefly, dissociated tumor cells from $ERG^{+}/PTEN^{-}$ mouse prostate tumors were injected subcutaneously with

Matrigel:PBS (1:1) in NRG (NOD-Rag2-IL2rg^{Tm1}/Rj) mice for two consecutive generations. After this step, tumor cells freshly isolated from the tumor xenografts were plated in cell culture dishes in DMEM medium (61965-026, Gibco®) supplemented with 10% FBS (FBS, Biowest, cat. S181B) and 1% Pen/Strep(Thermo Fisher, cat. 15070063). The established cell line (EPG2) was maintained in DMEM medium and regularly checked for mycoplasma contamination using the MycoAlert Mycoplasma detection kit.

Reagents

The σ1R ligands WMS 26-09, WMS 26-10, and WMS 26-02 were synthesized in Wünsch's laboratory and previously described (1). The compounds were dissolved in DMSO (10 mM) and stored at -20°C. For additional information, see the Reagents and Resources Table available online within the supplemental information file.

Mice

All animal studies were approved by the Swiss Veterinary Authority (license 32174; TI 04/2020). Male 4-week-old athymic nude mice purchased from Invigo were used to establish subcutaneous xenograft models with the indicated cell lines. NOD.Cg-*Prkdc*^{scid} *Il2rg*^{tm1Wjl}/SzJ (NSG) male mice were purchased from Charles River Laboratories and used to maintain patient-derived xenografts (PDXs) by serial passaging. Freshly dissociated cells from PDXs were cultured in tumor sphere-forming conditions. Transgenic/knockout *Pb-Cre4;Pten*$^{flox/flox}$*;R26*^{ERG} (ERG^+/$PTEN^-$) mice with combined prostate-specific deletion of PTEN and overexpression of ERG (2) were generated in-house and used for producing tumor spheres and organoids for *ex vivo* assays and for carrying out *in vivo* systemic treatment.

Molecular dynamics simulation

The starting structure for the σ1R was obtained from the RCSB Protein Data Bank (PDB ID 5HK1) (3). Only the protomer with the complete sequence was retained for the Molecular Dynamics simulations. The antechamber program from AMBER21 was used to assign atom types to each ligand (4). Docking and classical Molecular Dynamics simulations on σ1R in complex with WMS 26-09 and WMS 26-10 were carried out according to a well-validated procedure (1,5,6). Briefly, the system density and volume were relaxed in the NPT ensemble, maintaining the Berendsen barostat for 20 ns. After this step, 50 ns of unrestrained NVT production simulation was run for each system. All images were created using the UCSF Chimera software (7). Calculation of the free energy of binding (ΔG_{bind}) was performed via the Molecular Mechanics/Poisson Boltzmann Surface Area (MM/PBSA) approach.

Cell proliferation

DU145, PC3, 22RV1, and EPG2 ($2x10^3$ cells/well), VCaP (10^4 cells/well), and RWPE-1 ($5x10^3$ cells/well) were plated in 96-well plates and treated 24 h later with σ1R ligands. Viable cell number was measured after 72 h by staining cells with sulforhodamine B. Assays were repeated at least three times in independent experiments with three technical replicates in each experiment.

Tumor-sphere assay

Cells were seeded at a density of 10^3 cells/mL in 6-well tissue culture plates coated with Poly-(2-hydroxyethyl methacrylate) (Poly-HEMA, Sigma-Aldrich) in DMEM-F12 1:1 media (Gibco) containing 1x B-27 supplement (Invitrogen), 4 µg/mL insulin (I-6634, Sigma-Aldrich), 20 ng/mL FGF2 (F0291, Sigma-Aldrich), 10 ng/mL EGF (E9644, Sigma-Aldrich), 5% Pen/Strep (Invitrogen). Tumor spheres were counted after 10 days using a microscope. For drug treatment, σ1R ligands or vehicle were added to the culture medium. Assays were repeated three itimes independently with three technical replicates. For ex vivo assay from patient-derived tumor and PCa cell lines xenografts, and $ERG^+/PTEN^-$ prostate tumors, tissue was collected and dissociated into single-cell suspensions in Hank's buffered salt solution (Invitrogen) containing collagenase III (1 mg/mL, Worthington Biochem). The resulting cell suspension was filtered through 40-µm nylon mesh, and single cells were harvested and plated in vitro in anchorage-independent tumor-sphere forming conditions. *Ex-vivo* tumor-sphere assays were performed for all the mice in the in vivo experiment with three technical replicates per mouse.

Tumor organoid assay

For mouse-derived organoid generation, single cells were obtained *ex vivo* by mechanical dissociation and tissue digestion of prostatic tumor tissue from $ERG^+/PTEN^-$ mice or *in vitro* cultured EPG2 cells. Single cells were counted, and viable cells ($5x10^3$/sample) were resuspended and seeded in 10 µL of prostate culture medium (MPCM) combined with Matrigel (20%:80%). For drug treatment, vehicle or σ1R ligands were added to the 10 µL domes at the plating time.

In vivo tumor xenografts

DU145, PC3, and 22Rv1 ($3x10^6$ cells) were injected subcutaneously into the flank of 4-week-old male athymic nude mice (n=5). Growth of cell line-derived subcutaneous tumor xenografts was monitored with a caliper

three times a week until the end of the study. Tumor volumes were calculated using the formula (width×length^2)/2. At the end of the experiment, tumors were excised, and fresh tumor tissue was used for *ex vivo* tumor-sphere forming assays. To assess *in vivo* tumorigenicity of PDX-derived cells, tumor-sphere cells (10^4 cells/mouse) or bulk tumor cells (10^6 cells/mouse) from each PDXs were injected subcutaneously into the flank of 4-week-old male NSG mice (n=5).

Treatment of ERG$^+$/PTEN$^-$ mice

The *Pb-Cre4;Pten$^{flox/flox}$;R26^{ERG}* (ERG$^+$/PTEN$^-$) mice (38 week-old) were treated for four consecutive days with the σ1R ligand WMS 26-02 (40mg/kg, n=4) or vehicle (n=3) administered by intraperitoneal injection. At the end of the experiment, vehicle and drug-treated mice were euthanized and prostates dissected. Portions of the prostates were immediately processed to dissociate tumor cells and proceed *ex vivo* with tumor organoid assays as described above. The remaining portions of prostatic tissue were formalin-fixed and paraffin-embedded for immunohistochemistry.

Confocal microscopy

For confocal microscopy imaging, cells (2x10^4 cells/well) were seeded in 8-well Millicell EZ SLIDE (Ibidi) and treated with σ1R ligands for the indicated time. MitoTrackerGreen (cat. M7514; Life Technology), tetramethylrhodamine methyl ester (TMRM) (cat. T668; Life Technology), and ER tracker (cat. E34250; Life Technology) were added to the culture medium for 40 min at the end of the incubation according to the manufacturer's instructions. Cells were stained with an anti-σ1R antibody (Sigma-Aldrich HPA024071). Nuclei were stained with Hoechst 33342 (Thermo Fischer, 62249). Fluorescence images of adherent cells and tumor-sphere cells were obtained with live cells using a Leica TCS SP5 confocal laser microscope, acquiring pictures with a 63X/1.44 HCX PL APO CS objective. For mitochondria morphology analysis, cells were stained with MitoTrackerGreen. We acquired 5 to 13 independent images (z-step planes of 0.7 μm) per sample. For each condition, a total of 50 to 150 cells were examined. The Mitochondrial Network Analysis (MiNA) plugin of Fiji ImageJ was used to determine multiple parameters defining the state of the mitochondrial network morphology in individual cells, including the mean mitochondrial branch length, network branch mean, mean summed branch length, and mitochondrial footprint (8). The following parameters were applied: median filter: 2; unshaped mask: 3 and 0.1; CLAHE: 16, 100, and 3. MitoHacker was used to determine the mitochondrial footprint and other parameters.

Transmission electron microscopy

Cells were fixed with 2.5% glutaraldehyde in 0.1 M cacodylate buffer pH 7.4 for 1 h at room temperature. After several washes in cacodylate buffer, samples were postfixed with reduced osmium solution (1% OsO4, 1.5% potassium ferrocyanide in 0.1 M cacodylate buffer pH 7.4) for 2 h on ice. After several washes in milli-Q water, sections were incubated in 0.5% uranyl acetate overnight at 4°C. Samples were dehydrated with ethanol at increasing concentrations, embedded in epoxy resin, and polymerized for 48 h at 60°C. Ultrathin sections were obtained using an ultramicrotome (UC7, Leica microsystem, Vienna, Austria), collected on copper or nickel grids, and stained with uranyl acetate and Sato's lead solutions. Samples were examined in a Transmission Electron Microscope Talos L120C (FEI, Thermo Fisher Scientific) operating at 120kV. For quantitative mitochondria analysis, images acquired at 5300x were examined using Microscope Image Browser (MIB) software to evaluate parameters defining the mitochondria size and shape, including the length of the major mitochondrial axis (9).

Oxygen consumption rate

The Seahorse XFp Analyzer (Agilent-Seahorse Bioscience) was used to measure the mitochondrial oxygen consumption rate (OCR) in adherent cultures and tumor spheres. Adherent DU145 cells ($5x10^3$ cells/well) were seeded in Seahorse miniplates 24 h before the OCR measurement. Tumor sphere-derived cells ($1.5x10^4$ cells/well) were seeded in Cell-Tak-coated miniplates on the day of the OCR measurement. The MitoStress test was performed by pre-incubating cells in XF base medium supplemented with 1 mM pyruvate, 2 mM glutamine, and 10 mM glucose in a non-CO_2 incubator. OCR was measured under basal conditions and following the step-wise addition of oligomycin (1 µM), carbonyl cyanide-p-trifluoromethoxyphenylhydrazone (FCCP) (0.5 µM), and rotenone/antimycin A (0.5 µM). Data were normalized based on cell number by determining cell density using the sulforhodamine B assay (adherent cells) or the initial seeding cell count (tumor-sphere cells).

Quantitative RT-PCR

Cells and tissues were lysed with 500 µL TRI-Reagent (MGRGENE) and RNA was extracted using Direct-zol RNA MiniPrep kit (Zymo Research). Gene expression was determined by quantitative RT-PCR (qRT-PCR) using a One-Step qRT-PCR reaction (QuantiFast SYBR Green RT-PCR, Qiagen) and specific primers for σ1R on a StepOnePlus™ Real-Time PCR system (Life Technologies). β-Actin was used as a reference to normalize RNA levels. Data were analyzed with StepOne software v2.2 (Life Technologies). σ1R mRNA expression was detected using the forward primer 5'-TGTCCGAGTATGTGCTGCTC-3' and the reverse primer 5'-

AAGGTGCCAGAGATGATGGT-3’. The primer set for the β-actin gene was as follows: forward primer, 5’-GGTGCTCCATGAGGAGACA-3’, and reverse primer, 5’-CCTGCCTCTTTTCCACAGAA-3’. For qRT-PCR, samples from three independent experiments with three technical replicates were examined.

Immunoblotting and immunoprecipitation

Cell lysates were prepared using RIPA buffer (25 mM Tris-HCL, 150 mM KCL, 5 mM EDTA, 1% NP40, 0.1% SDS, 0.5% sodium deoxycholate) for 20 min at 4°C. Lysates were centrifuged at 10,000 rpm for 10 min at 4°C. Total proteins in the collected supernatants were quantified with a Pierce BCA protein assay kit (Thermo Scientific). Protein samples (30 µg) were separated by SDS-PAGE (12% PAGE for σ1R and 8% PAGE for β-catenin) and then transferred to PVDF membranes. After blocking for 10 min with 10% milk in TBS-T and washing three times for 5 min, membranes were incubated overnight with the primary antibody in TBS-T. After three washes of 5 min, membranes were incubated for 45 min at RT with secondary HRP-conjugated antibody (1:20000) in TBS-T. The signal was developed with Western Bright or Quantum ECL (Advasta-Witec). The blots were visualized using FUSION SOLO S, and band intensity was quantified with the equipment software. For immunoprecipitation, cell lysates prepared in RIPA buffer were incubated overnight at 4°C using ProteinG/proteinA-Agarose (IP05, Merk) with anti-β-catenin antibody or anti-Sigma1R antibody conjugated to agarose beads (sc-137075 AC, Santa Cruz). Immunoblotting was done as described above.

Immunohistochemistry

Tissue sections from archival formalin-fixed paraffin-embedded tissue specimens of primary prostate tumors (*n*=10) and hormone-refractory metastatic tumors (n=27) were obtained from Multimedica (Milan, Italy) upon approval by the Institutional Ethical Committee. After antigen retrieval, the tissue sections were incubated with anti-σ1R (Sigma-Aldrich, HPA020471) and anti-β-catenin antibodies at room temperature for 1h. For immunohistochemistry, incubation with the primary antibody was followed by biotinylated secondary antibody (Histostain Plus, Invitrogen) and streptavidin-horseradish peroxidase conjugate. The primary antibody was detected using 3-3’diaminobenzidine (DAB) chromogen. Cell nuclei were counterstained with a hematoxylin solution. Slides were scanned and digitalized using a Leica Aperio AT2 Scanner and the intensity of immunostaining was evaluated using Aperio ImageScope (v12.4.3.5008) software. Selected regions of interest (ROIs) were manually annotated and then analyzed by the software.

RNA sequencing

RNA was isolated from adherent cultures and tumor-spheres of DU145 cells in three independent replicates, marked with Illumina total prep 96 RNA amplification kit (Ambion). RNA sequencing for all experiments was performed using Next Ultra II Directional RNA Library Prep Kit for Illumina starting from 800 ng of total RNA from each sample and sequenced on the Illumina NextSeq500 with single-end, 75 base pair long reads. Sequencing reads were aligned to the GRch38.p12 release of the human genome and quantified using STAR aligner (10). RNA-Seq analysis was carried out using the DESeq2 pipeline (11). The Benjamini-Hochberg multiple-test correction method was adopted. RNA sequencing experiments were performed with three biological replicates per condition.

Protein extraction and enzymatic digestion for proteomic analysis

The proteomic analysis was performed with adherent DU145 cells. Three replicate samples were processed independently for each condition. Cell pellets of $2x10^6$ cells were produced and washed twice in phosphate-buffered saline (PBS), and dry pellets were flash-frozen and stored at -80°C. Cell lysis and protein extraction were performed by suspending each cell pellet in 50 µL of 8M urea in 50 mM ammonium bicarbonate (ABC) and sonicated for 15 min (Disruptor, Diagenode). Proteins (100 µg) were reduced with 10 mM dithiothreitol for 20 min at room temperature and alkylated with 50 mM iodoacetamide for 30 min at room temperature. Protein digestion was carried out in 8M urea, 50 mM ABC, for 2 h at room temperature with 1 µg of LysC, after which the digestion buffer was diluted with 50 mM ABC to final 2 M urea and 1 µg of trypsin was added for overnight digestion at room temperature. Digestion was arrested by adding acetonitrile (ACN) to 2% and trifluoroacetic acid (TFA) to 0.3% and the samples were cleared by centrifugation for 5 min at maximum speed. The supernatant was then loaded onto C18 StageTips, from which purified peptides were eluted with 80% ACN, and 0.5% acetic acid. Finally, the elution buffer was eliminated by vacuum centrifugation and purified peptides were resolved in 2% ACN, 0.5% acetic acid, and 0.1% TFA. For LC-MS/MS analysis, 1 µg of purified peptides from each sample were injected as single-shot measurements. Proteomics analysis by mass spectrometry was performed on three biological replicates per condition.

LC-MS/MS analysis

Peptides were separated on an EASY-nLC 1200 HPLC system (Thermo Fisher Scientific) coupled online to a Q Exactive HF mass spectrometer (Thermo Fisher Scientific) via a nanoelectrospray source (Thermo Fisher Scientific). Peptides were loaded in buffer A (0.1% formic acid) into a column (75 µm inner diameter, 50 cm length) in house packed with ReproSil-Pur C18-AQ 1.9 µm resin (Dr. Maisch HPLC GmbH), and eluted over

a 150-min linear gradient of 5-30% buffer B (80% ACN, 0.1% formic acid) at a flow rate of 250 nl/min. The Q Exactive HF was operated in a data-dependent mode with a survey scan range of 300-1,650 m/z, resolution of 60,000 at 200 m/z, maximum injection time of 20 ms, and AGC target of 3e6. Up to the ten most abundant ions with charge 2 to 5 were isolated with a 1.8 m/z isolation window and subjected to higher-energy collisional dissociation (HCD) fragmentation at a normalized collision energy of 27. MS/MS spectra were acquired with a resolution of 15,000 at 200 m/z, a maximum injection time of 55 ms, and an AGC target of 1e5. Dynamic exclusion was set to 30 seconds to reduce repeated sequencing. Data were acquired with the Xcalibur software (Thermo Fisher Scientific).

Proteomic data analysis

Xcalibur raw files were processed using the MaxQuant software v.1.6.7.0 (12). Searches were performed against the Human UniProt database (June 2019) and a common contaminants database by the integrated Andromeda search engine (13) to identify peptides and proteins with a false discovery rate of <1%. Enzyme specificity was set as “Trypsin/P” with a maximum of 2 missed cleavages and 7 as the minimum length required for peptide identification. N-terminal protein acetylation and methionine oxidation were set as variable modifications, and cysteine carbamidomethylation was set as a fixed modification. Match between runs was enabled to transfer identifications based on mass and normalized retention times, with a matching time window of 0.7 min and an alignment time window of 20 min. Label-free protein quantification (LFQ) was performed with the MaxLFQ algorithm, where a minimum peptide ratio count of 1 was required for quantification (14).

Downstream analysis of proteomic and transcriptomic data

All the following bioinformatic analyses were performed in the R environment (15). Proteomic data were processed according to the DEP pipeline (16). Data were first filtered according to the “Reverse”, “Potential contaminant”, “Only identified by site” criteria, and according to the presence of missing values: only the proteins quantified in at least 2 samples per condition were considered. Then, the normalization through the variance stabilizing transformation method and the imputation of missing values using a deterministic minimal value approach were performed. The differentially expressed proteins were obtained through a differential enrichment test based on protein-wise linear models and empirical Bayes statistics, and the Benjamini-Hochberg multiple-test correction method was applied. Volcano plot and heatmap images were made with pheatmap and plot R functions. Enrichment analysis was performed with the cameraPR function from the limma package (17). The accounted genes and proteins were weighted (statistic parameter) according to the Wald statistic (from “results” function by DESeq2 package), and the log2FoldChange respectively. Gene

expressions coming from patient datasets were weighted proportionally to the correlation with σ1R. To functionally annotate the lists of genes or proteins, the enrichR package was adopted (18). The synthesized gene ontology functional annotation results were obtained through the rrvgo package (19). In the case of multiple comparisons, the Benjamini-Hochberg correction method was used. The network analysis was performed with the Cytoscape software platform (20). The network was built considering as graph nodes all the proteins that were significantly (p-value < 0.05) differentially expressed with a log2FoldChange higher than 0.7 or lower than -0.7. To infer the interactions between the proteins (graph edges), the STRING database was adopted with the following set of parameters: confidence cutoff = 0.4 and maximum additional interactors = 0 (21). The network parameters were achieved through the "NetworkAnalyzer" tool.

Public genomic data analysis

We explored the correlation between the expression levels of σ1R gene (SIGMAR1) and the global gene expression profile, taking advantage of an integrated database of prostate cancer patients (22). The cohort consists of a total of 1106 samples, including 664 primary prostate cancers and 249 castration-resistant prostate cancers (CRPC). Technical replicates from the original database were excluded to spot biological variation without affecting statistical significance. For the analysis, we utilized variance stabilizing transformation (vst)-normalized expression data and applied Pearson's statistical testing. Gene set enrichment analysis (GSEA) was performed using the Clusterprofiler package (eps = 1e-50) (23). Gene-set collections were retrieved from the Molecular Signature Database (MsigDB). P-values were corrected for multiple testing using the False Discovery Rate (FDR).

Statistical analysis

Data are presented as mean ± SD. Tumor growths are presented as mean ± SEM. Replicates represent biologically independent samples. Differences between groups were assessed with an unpaired two-tailed *t*-test and one-way ANOVA and were considered statistically significant for $P < 0.01$.

Data availability

All data and materials that support the findings of this study are available within the article and supplemental information. Supplemental figures and datasets are available as supplemental information. RNA sequencing data reported in this study are deposited in the NCBI Gene Expression Omnibus (GEO, GSE203198). Any additional information required to reanalyze the data reported in this paper is available from the lead contact upon request.

REFERENCES

1. Kopp N, Civenni G, Marson D, Laurini E, Pricl S, Catapano CV*, et al.* Chemoenzymatic synthesis of 2,6-disubstituted tetrahydropyrans with high sigma1 receptor affinity, antitumor and analgesic activity. Eur J Med Chem **2021**;219:113443
2. Chen Y, Chi P, Rockowitz S, Iaquinta PJ, Shamu T, Shukla S*, et al.* ETS factors reprogram the androgen receptor cistrome and prime prostate tumorigenesis in response to PTEN loss. Nat Med **2013**;19:1023-9
3. Schmidt HR, Zheng S, Gurpinar E, Koehl A, Manglik A, Kruse AC. Crystal structure of the human sigma1 receptor. Nature **2016**;532:527-30
4. Wang J, Wolf RM, Caldwell JW, Kollman PA, Case DA. Development and testing of a general amber force field. J Comput Chem **2004**;25:1157-74
5. Kronenberg E, Weber F, Brune S, Schepmann D, Almansa C, Friedland K*, et al.* Synthesis and Structure-Affinity Relationships of Spirocyclic Benzopyrans with Exocyclic Amino Moiety. J Med Chem **2019**;62:4204-17
6. Kopp N, Holtschulte C, Borgel F, Lehmkuhl K, Friedland K, Civenni G*, et al.* Novel sigma1 antagonists designed for tumor therapy: Structure - activity relationships of aminoethyl substituted cyclohexanes. Eur J Med Chem **2021**;210:112950
7. Pettersen EF, Goddard TD, Huang CC, Couch GS, Greenblatt DM, Meng EC*, et al.* UCSF Chimera--a visualization system for exploratory research and analysis. J Comput Chem **2004**;25:1605-12
8. Valente AJ, Maddalena LA, Robb EL, Moradi F, Stuart JA. A simple ImageJ macro tool for analyzing mitochondrial network morphology in mammalian cell culture. Acta Histochem **2017**;119:315-26
9. Belevich I, Joensuu M, Kumar D, Vihinen H, Jokitalo E. Microscopy Image Browser: A Platform for Segmentation and Analysis of Multidimensional Datasets. PLoS Biol **2016**;14:e1002340
10. Alexander D. STAR: ultrafast universal RNA-seq aligner. Bioinformatics (Oxford, England) 2013 2013:15-21.
11. Anders S, Heidelberg E. DESeq: Differential gene expression analysis based on the negative binomial distribution. 2019.
12. Cox J, Mann M. MaxQuant enables high peptide identification rates, individualized p.p.b.-range mass accuracies and proteome-wide protein quantification. Nat Biotechnol **2008**;26:1367-72
13. Cox J, Neuhauser N, Michalski A, Scheltema RA, Olsen JV, Mann M. Andromeda: a peptide search engine integrated into the MaxQuant environment. J Proteome Res **2011**;10:1794-805

14. Cox J, Hein MY, Luber CA, Paron I, Nagaraj N, Mann M. Accurate proteome-wide label-free quantification by delayed normalization and maximal peptide ratio extraction, termed MaxLFQ. Mol Cell Proteomics **2014**;13:2513-26
15. Team RC. R: A Language and Environment for Statistical Computing. Vienna, Austria: R Foundation for Statistical Computing; 2020.
16. Smits A, Huber W. DEP: Differential Enrichment analysis of Proteomics data. 2020.
17. Smyth G, Hu Y, Ritchie M, Silver J, Wettenhall J, McCarthy D*, et al.* limma: Linear Models for Microarray Data. 2020.
18. Jawaid W. enrichR: Provides an R Interface to Enrichr. 2021.
19. Sayols S. rrvgo: a Bioconductor package to reduce and visualize Gene Ontology terms. 2020.
20. Shannon P, Markiel A, Ozier O, Baliga NS, Wang JT, Ramage D*, et al.* Cytoscape: a software environment for integrated models of biomolecular interaction networks. Genome research **2003**;13:2498-504
21. Jensen LJ, Kuhn M, Stark M, Chaffron S, Creevey C, Muller J*, et al.* STRING 8--a global view on proteins and their functional interactions in 630 organisms. Nucleic acids research **2009**;37:D412-6
22. Bolis M, Bossi D, Vallerga A, Ceserani V, Cavalli M, Impellizzieri D*, et al.* Dynamic prostate cancer transcriptome analysis delineates the trajectory to disease progression. Nat Commun **2021**;12:7033
23. Yu G, Wang LG, Han Y, He QY. clusterProfiler: an R package for comparing biological themes among gene clusters. OMICS **2012**;16:284-7

REAGENTS AND RESOURCES TABLE

REAGENT or RESOURCE	SOURCE	IDENTIFIER
**Antibodies**		
Rabbit polyclonal anti-Sigma1R	Sigma-Aldrich	HPA024071
Mouse monoclonal anti-Sigma1R	Santa Cruz	sc-137075
Rabbit polyclonal anti-MFF	Sigma-Aldrich	HPA010968
Mouse monoclonal anti-α-Tubulin	Calbiochem	CP06-100UG
Rabbit polyclonal anti-β-Catenin	Cell signaling	8480
Rabbit polyclonal anti-β-actin	Cell signaling	4967
Rabbit polyclonal anti-Androgen Receptor	Millipore	06-680
Rabbit monoclonal anti-Mitofusin-2	Abcam	ab124773
Rabbit monoclonal anti-DRP1	Abcam	ab184247
Mouse monoclonal anti-GAPDH	Santa Cruz	sc-47724
**Lentiviral particle expressing shRNA**		
Sigma1R	Sigma-Aldrich	SHCLNV-NM_005866
β-Catenin	Sigma-Aldrich	SHCLNV-NM_001904
Control	Sigma-Aldrich	SHC016
**Biological Samples**		
Archival FFPE tissue specimens	Multimedica, Italy	N/A
**Chemicals, Peptides, and Recombinant Proteins**		
MitoTracker Green	Life Technology	M7514
ER Tracker	Life Technology	E34250
TMRM	Thermo Scientific	T668
B-27 supplement	Life Technologies	17504044
7-AAD	Sigma-Aldrich	A9400
poly-HEMA	Sigma-Aldrich	H9268
Sulforhodamine B	Sigma-Aldrich	S1402
Puromycin dihydrochloride	Sigma-Aldrich	P8833
hEGF	Sigma-Aldrich	E9644
Insulin	Sigma-Aldrich	I6634
bFGF2	Sigma-Aldrich	F0291
Hexadimethrine bromide (polybrene)	Sigma-Aldrich	H9268
PRE-084	Sigma-Aldrich	P2607
DiHydro-Testosterone (DHT)	Sigma-Aldrich	A8380
RPMI 1640	Gibco	21875-034
DMEM	Gibco	61965-026
MEBM	Lonza	CC-3151
Collagenase Type 3	Worthington	4182
Matrigel Basement Membrane Matrix	Corning	354234
XenoLight D-Luciferin-K+ Substrate	PerkinElmer	122796
BI-5521	Boehringer Ingelheim	
PS341	Selleckchem	S1013
**Critical Commercial Assays**		
JetPrime®	Thermo Fisher	Cat. 114-15
XPs Cell Mito Stress Test Kit	Agilent Technologies	Cat.1033010
TRI-Reagent	MGRGENE	TR118
RNA amplification kit	Ambion	4393543
Direct-zol RNA MiniPrep kit	Zymo Research	R2052
Luciferase Assay System Kit	Promega	E1500
QuantiFast SYBR Green RT-PCR	Qiagen	204154
Pierce BCA protein assay kit	Thermo Scientific	23225
Western Bright	Advasta-Witec	K 12045-D45

Quantum ECL	Advasta-Witec	K 12045-D10
SYBR Green PCR Master Mix	Applied Biosystems	KK4605
**Oligonucleotides**		
5'-TGTCCGAGTATGTGCTGCTC-3'	Sigma-Aldrich	σ_1R-F qRT-PCR
5'-AAGGTGCCAGAGATGATGGT-3'	Sigma-Aldrich	σ_1R-R qRT-PCR
5'-GGTGCTCCATGAGGAGACA-3'	Sigma-Aldrich	β-actin-F qRT-PCR
5'-CCTGCCTCTTTTCCACAGAA-3'	Sigma-Aldrich	β-actin-R qRT-PCR
**Deposited Data**		
Raw gene expression data	This study	NCBI GEO: GSE203198
Gene expression data	Oncomine	www.oncomine.com
**Experimental Models: Cell Lines**		
DU145	ATCC	HTB-81
PC3	ATCC	CRL-1435
LNCaP	ATCC	CRL-1740
LNCaP-abl	Cellosaurus	CVCL-4793
LNCaP-C4-2	ATCC	CRL-3314
22Rv1	ATCC	CRL-2505
RWPE1	ATCC	CRL-11609
NCI-H660	ATCC	CRL-5913
VCaP	ATCC	CRL-2876
**Experimental Models: Organisms/Strains**		
Athymic mice	Charles River	N/A
NSG	Charles River	N/A
Pb-Cre4;Pten$^{flox/flox}$;R26^{ERG}		Shinde et al., 2019
LuCaP 145.2		Nguyen et al., 2017
LuCaP 35		Nguyen et al., 2017
**Recombinant DNA**		
GFP-σ1R	Origene	#RG201206
Human β-catenin N90-Myc	Addgene	#31785
**Software and Algorithms**		
GraphPad Prism Version 6	GraphPad	N/A
AlphaEaseFC software	Alpha Innotech	N/A
FlowJo 10.8.1 Software	FlowJo	N/A
StepOne software v2.2	Life Technologies	N/A
FUSION SOLO S software	Witec	N/A
ImageJ Fiji	NIH	https://imagej.nih.gov/ij/
BRB array tools	NIH	http://brb.nci.nih.gov/BRB-ArrayTools/
UCSC Genome Browser	UCSC	http://genome.ucsc.edu/
GenomeStudio	Illumina	
Enrichr	Kuleshov et al. 2016	http://amp.pharm.mssm.edu/Enrichr/
GSEA and MSigDB	Tamayo, et al. 2005	http://software.broadinstitute.org/gsea/msigdb
Bowtie	Langmead et al., 2009	https://sourceforge.net/projects/bowtie-bio/
**Other**		
8-well Millicell EZ SLIDE	Millipore	80826

SUPPLEMENTARY DATASETS

The Supplementary Datasets are provided as separate Excel files.

SI Dataset S1. Differential gene expression analysis after σ_1R knockdown in bulk adherent DU145 cells.

SI Dataset S2. Differential gene expression analysis after σ_1R knockdown in DU145 tumor-spheres.

SI Dataset S3. Differential protein expression analysis after σ_1R knockdown in bulk adherent DU145 cells.

SI Dataset S4. Genes correlated to σ_1R expression in primary and CRPC clinical samples.

SI Dataset S5. Proteins modulated by σ_1R knockdown without transcript changes in DU145 cells.

SI Dataset S6. Local network parameters for proteins deregulated in σ_1R-depleted DU145 cells.
